# Host-Adapted *Apilactobacillus kunkeei* and Yeast Co-Fermentation Improves Fermented Bee Pollen Quality and Physiological Performance in *Heterotrigona itama*

**DOI:** 10.3390/microorganisms14071415

**Published:** 2026-06-28

**Authors:** Narathip Kongsamret, Petcharat Ponpichai, Kittiya Khongkool, Supachai Nitipan, Monthon Lertworapreecha, Jakkrawut Maitip, Bajaree Chuttong, Wankuson Chanasit

**Affiliations:** 1 Faculty of Science and Digital Innovation, Thaksin University, Phatthalung 93210, Thailand; 2 Faculty of Science, Energy and Environment, King Mongkut’s University of Technology North Bangkok, Rayong 21120, Thailand; 3 Meliponini and Apini Research Laboratory, Faculty of Agriculture, Chiang Mai University, Chiang Mai 52000, Thailand

**Keywords:** Stingless bee, *Heterotrigona itama*, host-adapted probiotics, *Apilactobacillus kunkeei*, Fermented bee pollen, LAB–yeast co-fermentation

## Abstract

Host-adapted probiotics offer a promising strategy for improving stingless bee nutrition and colony sustainability. In this study, gut-derived lactic acid bacteria (LAB) isolated from *Heterotrigona itama* were evaluated for probiotic potential and used to develop fermented bee pollen. Of 37 presumptive LAB isolates, three strains (BP-2, BP-3, and BPW-B1) exhibited strong tolerance to simulated gastrointestinal conditions, favorable adhesion-related properties, and acceptable safety profiles. Phylogenetic and biochemical analyses identified the selected isolates as *Apilactobacillus kunkeei*. The LAB strains were co-cultured with the osmophilic yeasts *Zygosaccharomyces bailii* TSU_YK2 and *Starmerella meliponinorum* TSU_YP10 to establish a host-associated LAB–yeast co-fermentation model that mimics stingless bee pollen fermentation. Co-fermentation significantly improved protein digestibility, organic acid production, antioxidant activity, and microbial viability relative to spontaneous fermentation controls (*p* < 0.05). Feeding experiments demonstrated that probiotic-fermented pollen increased feed intake, body weight, abdominal lipid reserves, hypopharyngeal gland development, and survival among *H. itama* workers. In addition, probiotic supplementation was associated with shifts in the dominant gut-associated bacterial taxa, including *Lactobacillus*, *Bifidobacterium*, and *Snodgrassella*. This study demonstrates the potential of combining gut-derived *A. kunkeei* with osmophilic yeasts as a functional fermentation starter culture to develop biologically relevant probiotic feed supplements for stingless bees.

## 1. Introduction

Stingless bees (Meliponini) are important pollinators in tropical ecosystems and contribute substantially to agricultural productivity and biodiversity. Among them, *Heterotrigona itama* is one of the most economically important stingless bee species in Southeast Asia because of its adaptability and suitability for meliponiculture [1,2,3]. However, environmental stressors such as habitat disturbance, climate variability, and nutritional imbalance may impair colony development and physiological performance [4,5]. Nutritional stress is a major factor in bee health because inadequate or low-quality diets can impair larval development, reduce lifespan, increase susceptibility to pathogens, and cause environmental stress [6,7]. Therefore, improving nutritional function and microbial balance has become an important strategy to enhance stingless bee health and colony sustainability.

Bee pollen is the primary protein source for bees and contains essential amino acids, lipids, vitamins, minerals, and phenolic compounds required for larval growth and adult physiology [8,9,10,11]. In stingless bees, collected pollen undergoes natural fermentation within brood cells to form bee bread, driven by a diverse microbial consortium consisting primarily of lactic acid bacteria (LAB), yeasts, and filamentous fungi [12,13,14,15]. During fermentation, microbial activity improves nutrient bioavailability by partially degrading pollen walls and producing metabolites such as organic acids and antioxidants [16,17,18,19]. LAB-mediated acidification may also contribute to the preservation of fermented pollen and the suppression of undesirable microorganisms [20,21,22]. However, natural fermentation is highly variable and can lead to inconsistent nutritional quality, underscoring the need for controlled microbial fermentation systems for developing functional bee feed.

The gut microbiota of bees comprises specialized microbial communities that contribute to nutrient metabolism, carbohydrate fermentation, and maintenance of microbial balance in the gastrointestinal (GI) tract [23,24]. Core bacterial groups commonly associated with social bees include *Lactobacillus*, *Bifidobacterium*, and *Snodgrassella*, which are involved in carbohydrate utilization and organic acid production [24,25,26,27,28]. Among these microorganisms, *Apilactobacillus kunkeei* is a dominant fructophilic lactic acid bacterium (FLAB) frequently found in nectar, honey, bee bread, and the bee’s GI tract [29,30]. This species is highly adapted to sugar-rich environments and has been reported to produce antimicrobial metabolites, including organic acids, which may help maintain microbial balance and suppress pathogens in bees [16,29,31,32,33]. Previous studies have also suggested that probiotic supplementation may improve nutrient utilization, physiological condition, and gut microbial composition in honey bees and stingless bees [5,34,35,36,37]. Vitellogenin was selected as a nutritional physiology marker because it is closely associated with nutrient status, longevity, and physiological condition in social bees [38,39]. Nevertheless, many studies have focused primarily on LAB isolated from honey or bee bread rather than from the bee’s GI tract, which may not fully reflect host-adapted functionality and ecological compatibility within the host environment. In addition to LAB, osmophilic yeasts such as *Zygosaccharomyces* and *Starmerella* are commonly associated with stingless bee colonies and contribute to carbohydrate metabolism and pollen fermentation [13,15,40]. Interactions between LAB and yeasts may enhance fermentation efficiency through complementary metabolic activities, creating opportunities to develop host-associated co-fermentation systems for producing functional bee feed [10,19,20,40,41].

Despite growing interest in bee-associated probiotics, studies integrating gut-derived LAB, controlled pollen fermentation, and in vivo evaluation in stingless bees remain limited. In particular, the functional interactions between host-adapted LAB and osmophilic yeasts during bee pollen fermentation remain poorly understood. Therefore, this study aimed to isolate and characterize the gut-derived *Apilactobacillus kunkeei* from *Heterotrigona itama*, develop a naturally associated LAB–yeast co-fermentation system for fermented bee pollen, and evaluate its effects on the functionality of fermented bee pollen and the physiological performance of *H. itama* workers.

## 2. Materials and Methods

### 2.1. Isolation and Screening of Gut-Derived Lactic Acid Bacteria from Heterotrigona itama

Worker stingless bees of *Heterotrigona itama* were collected from five geographically distinct locations that represent different environmental conditions (Table S1). Approximately 100 workers from three randomly selected colonies at each location were pooled before homogenization to obtain sufficient microbial biomass and reduce individual variability. Before dissection, bee surfaces were sterilized with 95% (*v*/*v*) ethanol. GI tracts were aseptically dissected, and approximately 0.1 g of pooled gut tissue was homogenized under sterile conditions. Lactic acid bacteria (LAB) were isolated using a modified method from Goh et al. (2021) [1]. Briefly, homogenized samples were serially diluted and enriched in De Man, Rogosa, and Sharpe (MRS) broth (Merck, Darmstadt, Germany) under anaerobic conditions at 35 ± 2 °C for 48 h. Enriched cultures were further diluted in sterile 0.85% NaCl and spread onto MRS agar supplemented with 0.01% (*w*/*v*) sodium azide to suppress Gram-negative bacteria. After anaerobic incubation at 35 ± 2 °C for 48 h, distinct colonies were purified and preliminarily characterized by Gram staining, cell morphology, catalase activity, and endospore formation [42].

### 2.2. Probiotic Properties Assessment

#### 2.2.1. Viability of LAB Under Simulated Gastrointestinal Conditions

Although stingless bees lack mammalian gastrointestinal physiology, this assay was adapted as a comparative stress-tolerance model commonly used in bee-associated probiotic studies [43,44]. LAB isolates were cultured in MRS broth for 48 h, harvested by centrifugation (7660× *g*, 15 min, 4 °C), washed twice with sterile 0.85% NaCl, and resuspended in sterile 50% (*w*/*v*) sucrose. Cell density was adjusted to OD_600_ = 1.0 ± 0.05. The standardized suspension was exposed to simulated gastric solution containing 0.85% NaCl, 100 U/mL α-amylase, and 0.15% pepsin (pH 3.0), and incubated at 35 ± 2 °C for 2 h with gentle agitation (80 rpm). Subsequently, cells were transferred to simulated intestinal solution containing 0.85% NaCl, 0.1% pancreatin, and 0.3% bile salts (pH 7.5) and incubated for an additional 2 h under the same conditions. Viable cell counts were determined at 0 h, after gastric exposure, and after intestinal exposure, and were expressed as log CFU/mL.

#### 2.2.2. Cell Surface Properties

LAB isolates were cultured in MRS broth at 35 ± 2 °C for 12 h. Cells were harvested by centrifugation (7660× *g*, 15 min, 4 °C), washed twice with sterile 0.85% NaCl, and standardized to approximately 1 × 10^8^ CFU/mL.

Auto-aggregation (AA) was evaluated according to Cozzolino et al. (2020) [45] by measuring OD_580_ after 4 h of incubation and calculated as:

AA (%) = [1 − (OD_t_/OD_0_)] × 100

where OD_0_ represents the initial optical density at time 0, and OD_t_ represents the optical density after incubation.

Cell surface hydrophobicity was determined using the bacterial adhesion to hydrocarbons (BATH) method with xylene and toluene as described by Iorizzo et al. (2020) [46]. Equal volumes of bacterial suspension and solvent were mixed and allowed to separate, after which the absorbance of the aqueous phase was measured at 580 nm. Hydrophobicity was then calculated as:

Hydrophobicity (%) = [1 − (OD_t_/OD_0_)] × 100

where OD_0_ represents the initial optical density of the bacterial suspension, and OD_t_ represents the optical density after phase separation.

Co-aggregation ability was evaluated using *Escherichia coli* ATCC 25922 and *Serratia marcescens* ATCC 13880 as indicator strains following Lee et al. (2024) [22]. Equal volumes of LAB and pathogen suspensions were mixed and incubated at 35 ± 2 °C for 4 h. The co-aggregation percentage was calculated as:

Co-aggregation (%) = {[(A_x_ + A_y_)/2 − A_(x+y)_]/[(A_x_ + A_y_)/2]} × 100

where A_x_ and A_y_ are the absorbances of the individual bacterial suspensions, and A_(x+y)_ is the absorbance of the mixed suspension after incubation.

These assays were used as indirect indicators of adhesion-related probiotic properties.

### 2.3. Safety Profiling

Safety assessment of LAB isolates was performed using hemolytic activity and antibiotic susceptibility testing. Hemolytic activity was evaluated by streaking overnight cultures onto blood agar supplemented with 5% (*v*/*v*) sheep blood, then incubated at 35 ± 2 °C for 48 h under anaerobic conditions. Hemolysis patterns were classified as α-, β-, or γ-hemolysis following Albanese et al. (2026) [34]. The absence of β-hemolysis was considered indicative of probiotic safety. Antibiotic susceptibility was determined by the Kirby–Bauer disk diffusion method [34]. Approximately 100 µL of LAB suspension (1 × 10^8^ CFU/mL) was spread on MRS agar plates, followed by application of antibiotic discs. Plates were incubated at 35 ± 2 °C for 24 h, and inhibition zones were measured. Results were interpreted according to CLSI guidelines as susceptible (S), intermediate (I), or resistant (R).

### 2.4. Coexistence Test

Co-cultivation compatibility among selected LAB strains was evaluated as described by Guo et al. (2009) [47]. Each strain was cultured under optimal growth conditions and streaked perpendicularly onto MRS agar plates, maintaining approximately 5 mm between streak lines. The plates were incubated at 35 ± 2 °C for 24–48 h and then examined for inhibition zones or other antagonistic interactions among strains.

### 2.5. Identification of LAB Isolates

Molecular identification of selected LAB isolates was performed by partial 16S rRNA gene sequencing. Genomic DNA was extracted using the G-spin™ Total DNA Extraction Kit (iNtRON, Biotechnology Inc., Seongnam-si, Republic of Korea) following the manufacturer’s instructions. The 16S rRNA gene was amplified with universal primers 27F and 1492R [1,3]. PCR products were verified by 1% agarose gel electrophoresis and sequenced by Macrogen Inc. (Seoul, Republic of Korea). Obtained sequences were compared with the NCBI nucleotide database using BLASTn (2.16.0). Multiple sequence alignments were performed using Clustal X and MEGA11. Sequences with ≥99% similarity were considered closely related species, and representative sequences were deposited in GenBank. Phylogenetic analysis was conducted using the neighbor-joining method with 1000 bootstrap replicates. Selected isolates were further characterized using API ZYM and API 50CHL systems (bioMérieux SA, Marcy-l’Étoile, France) to evaluate enzymatic activity and carbohydrate utilization profiles.

### 2.6. Solid-State Fermentation of Bee Pollen

Selected probiotic LAB and yeast strains were cultivated separately before fermentation. LAB strains were cultured in modified MRS broth according to Di Cagno et al. (2019) [14], while yeast strains, identified as *Zygosaccharomyces bailii* TSU_YK2 (PZ113379) and *Starmerella meliponinorum* TSU_YP10 (PZ113380), were cultured in Potato Dextrose Broth (PDB). Both yeast strains were originally isolated from the bee bread of *Heterotrigona itama* colonies. After incubation, cultures were harvested by centrifugation (2990× *g*, 10 min, 4 °C), washed twice with phosphate-buffered saline (PBS), and standardized to approximately 10^8^ CFU/mL for LAB and 10^7^ CFU/mL for yeasts. Starter formulations were prepared as shown in Table S2. Multifloral bee pollen (Fora Bee^®^, Chiangmai Healthy Product Co., Ltd., Chiang Mai, Thailand), predominantly derived from *Mimosa pudica*, was used as the substrate for solid-state fermentation following modified methods described in previous studies [14,17,18]. Briefly, 100 g of bee pollen was inoculated with LAB and yeasts to achieve final concentrations of approximately 10^7^ CFU/g and 10^6^ CFU/g, respectively, at a 1:1 inoculum ratio. Moisture content was adjusted to 40–50% (*w*/*w*) with sterile distilled water. Fermentation was carried out under static solid-state conditions at 30 °C for 14 days, followed by four days of air drying. The fermented product was then stored at 4 °C until further analysis. A spontaneous fermentation control was prepared by replacing the microbial inocula with sterile distilled water while maintaining identical fermentation conditions. Raw, unfermented pollen served as the baseline reference for physicochemical and functional analyses. However, LAB-only fermentation was not included as an independent treatment because the study was designed to evaluate LAB–yeast co-fermentation that mimics the natural microbial ecology of stingless bee pollen fermentation. In this ecology, osmophilic yeasts such as *Zygosaccharomyces* and *Starmerella* are core microbial members associated with stingless bee pollen, honey pots, and bee bread, where they contribute to nutrient transformation, sterol-related compound production, and fermentation stability [13,15,40,41].

### 2.7. Physicochemical Properties of Fermented Bee Bread

#### 2.7.1. Protein Content and In Vitro Protein Digestibility

The protein content of fermented bee bread was determined according to AOAC (2000) methods and expressed on a dry-weight basis. Soluble protein was quantified using the Bradford assay. For in vitro protein digestibility, 1 g of dried fermented pollen was suspended in 100 mL of 0.0075 N HCl containing 0.002% pepsin and incubated at 45 °C for 16 h with continuous shaking. After incubation, samples were filtered, and soluble protein content was quantified by the Bradford method using bovine serum albumin (BSA) as the standard. Absorbance was measured at 595 nm, and protein digestibility was expressed as the percentage of soluble protein released relative to total protein content [8,17].

#### 2.7.2. Amino Acid Profile

The amino acid composition of fermented bee bread was analyzed by HPLC (Agilent 1100, Waldbronn, Germany) equipped with a fluorescence detector and AccQTag column (3.9 × 150 mm). Samples were hydrolyzed with 6 N HCl at 110 °C for 22 h, filtered through a 0.45 μm membrane, derivatized, and chromatographically separated at 37 °C using AccQTag Eluent A and 60% acetonitrile as the mobile phase at a flow rate of 1.0 mL/min. Amino acids were detected at excitation and emission wavelengths of 250 and 395 nm, respectively, and reported as mg/g dry sample.

#### 2.7.3. Total Acidity and Organic Acid Analysis

Total acidity was determined as described by Dranca et al. (2020) [9]. Briefly, 2 g of fermented bee bread was homogenized with 5 mL Milli-Q water and titrated with 0.05 M NaOH to pH 8.5. Organic acids were analyzed using GC–MS (Thermo Scientific TRACE™ 1310 GC coupled with a single quadrupole mass spectrometer, Thermo Fisher Scientific, Waltham, MA, USA) following Lee et al. (2021) [48]. Briefly, dried samples were derivatized with pyridine and BSTFA containing 1% TMCS, then analyzed on a DB-5MS capillary column using helium as the carrier gas. Organic acids were identified by comparing retention times and mass spectra against reference standards and the NIST mass spectral library.

#### 2.7.4. Determination of Antioxidant Activity

DPPH radical-scavenging activity was determined as described by Lee et al. (2021) [48] and Urcan et al. (2024) [18], with minor modifications. Fermented pollen samples were extracted with 5% perchloric acid and centrifuged before filtration through a 0.45 μm membrane. An aliquot of the sample extract was mixed with 0.2 mM DPPH solution and incubated at 25 °C in the dark for 30 min. Absorbance was measured at 517 nm using ascorbic acid as the reference antioxidant. DPPH scavenging activity was calculated as:

DPPH scavenging activity (%) = [(A_blank_ − A_sample_)/A_blank_] × 100

where A_blank_ represents the absorbance of the control and A_sample_ represents the absorbance of the sample extract.

#### 2.7.5. Microbial Counts During Bee Bread Fermentation

Microbial populations during bee bread fermentation were enumerated on MRS agar for LAB and on Potato Dextrose Agar (PDA) for yeasts. Samples collected on days 0, 7, and 14 were serially diluted in sterile PBS before plating. MRS agar was supplemented with cycloheximide to suppress fungal growth, and PDA was supplemented with chloramphenicol to inhibit bacterial growth. Plates were incubated at 30 °C, and microbial counts were expressed as log CFU/g [14,17].

### 2.8. Feeding Trial to Evaluate Probiotic Performance in Heterotrigona itama

#### 2.8.1. Diet Preparation

Four dietary treatments were prepared to evaluate the effects of probiotics on *Heterotrigona itama*. Diet I: 50% (*w*/*v*) sucrose solution and served as the control. Diet II: basal multifloral bee pollen (Fora Bee^®^) without microbial supplementation. Diet III: bee pollen supplemented with selected yeast strains (*Zygosaccharomyces bailii* TSU_YK2 and *Starmerella meliponinorum* TSU_YP10) at a final concentration of approximately 10^5^ CFU/g. Diet IV: selected LAB (10^6^ CFU/g) combined with yeast strains (10^5^ CFU/g). The total inoculum concentration was adjusted to 10% (*v*/*w*). All diets were prepared using 100 g of pollen substrate and stored at 4 °C until use.

#### 2.8.2. Cage Experiment

The feeding experiment was conducted with three healthy queenright colonies of *Heterotrigona itama* maintained at the Pantae Community Enterprise in Khuan Khanun District, Phatthalung province, Thailand (locality: 7.806259568725061, 100.01692785193286). The colonies were visually inspected to confirm the absence of parasites or disease symptoms and to ensure adequate supplies of pollen and honey reserves. To obtain newly emerged workers of similar age, brood combs were carefully removed from each colony and incubated in plastic containers (500 cm^3^) in the dark at 28 ± 2 °C and 70 ± 10% relative humidity (RH) to simulate natural hive conditions [49]. Newly emerged workers without visible abnormalities were randomly assigned to hoarding cages (200 cm^3^) [2]. A total of 500 newly emerged workers were equally distributed across 20 cages (25 bees per cage), with individuals from all three colonies to minimize colony-specific bias and genetic variation. Nevertheless, because the workers originated from a limited number of colonies and were subsequently redistributed among cages, colony-level effects could not be entirely ruled out. Five replicate cages were assigned to each dietary treatment: Diet I (50% sucrose control), Diet II (basal pollen), Diet III (yeast supplementation: *Zygosaccharomyces bailii* TSU_YK2 and *Starmerella meliponinorum* TSU_YP10), and Diet IV (LAB combined with yeasts). All cages were supplied ad libitum with 50% (*w*/*v*) sucrose solution as a carbohydrate source [4]. Fermented pollen diets were provided using 2.5 mL Eppendorf feeders and replaced every four days to minimize microbial contamination [50]. Feeding trials were conducted for 14 days under laboratory conditions to evaluate the effects of probiotic supplementation on the physiological performance of *H. itama*.

##### Feed Intake and Body Weight Change

Feed intake and body weight changes were monitored throughout the 14-day feeding period and calculated using the methods described by Straub et al. (2024) and Williams et al. (2013) [49,50].

Average daily intake (g/bee/day) = total diet consumed (g)/(number of bees × number of days)

Body weight change (g/bee) = Final body weight − Initial body weight

##### Abdominal Lipid Content

Abdominal lipid content (ALC) was determined using the ether extraction method described by Güneşdoğdu et al. (2024) [51]. Ten adult workers per treatment were dissected, and abdomens were dried at 45 °C for 72 h to obtain the initial dry weight (IDW). Samples were extracted with ethyl ether for 24 h, then re-dried to obtain the final dry weight (FDW). Lipid content was calculated as:

ALC (mg/g) = [(IDW − FDW)/IDW] × 1000

##### Survival Rate

Survival rate was assessed by counting the number of surviving bees at the end of the feeding trial according to Kim et al. (2024) [52] using the following equation:

Survival rate (%) = (Ns/N_0_) × 100

where N_s_ represents the number of surviving bees, and N_0_ represents the initial number of bees.

##### Hypopharyngeal Gland Development

Hypopharyngeal glands (HPGs) were dissected from workers in each treatment group (*n* = 10 bees per treatment). HPG acini size was measured on day 14 post-treatment, with approximately 30 acini measured per bee following Straub et al. (2024) [49]. For protein analysis, dissected glands were homogenized in phosphate buffer (pH 7.8), centrifuged, and the supernatant collected for protein quantification by using the Bradford method. Protein content was expressed as mg protein/g tissue, following Harwood et al. (2019) [39].

##### Gene Expression and Vitellogenin Quantification by qPCR

For gene expression analysis, approximately 10 adult workers per replicate were pooled and flash-frozen in liquid nitrogen prior to RNA extraction. Total RNA was extracted using the RNeasy Mini Kit (Qiagen, Hilden, Germany) following the manufacturer’s instructions, and RNA concentration was measured using the Qubit RNA BR Assay Kit (Thermo Fisher, Thermo Fisher Scientific, Waltham, MA, USA). Complementary DNA (cDNA) was synthesized with a commercial reverse transcription kit (Promega, Promega Corporation, Madison, WI, USA). Negative controls were included in the RNA extraction and reverse transcription procedures. Expression of the nutrition-related gene vitellogenin (Vg) was evaluated using previously reported primers [53]: Vit_f (GCAGAATACATGGACGGTGT) and Vit_r (GAACAGTCTTCGGAAGCTTG), yielding a 146 bp amplicon. Relative gene expression levels were normalized against β-actin and RPS5 reference genes and expressed as arbitrary units (AU) [38]. Quantitative real-time PCR (qPCR) was performed using the QuantiTect SYBR Green PCR Kit (Qiagen, Hilden, Germany) on a StepOne Real-Time PCR System (Applied Biosystems, Foster City, CA, USA). Each 20 µL reaction contained 1× SYBR Green Master Mix, 0.5 µM of each primer, 5 µL of diluted cDNA template, and RNase-free water. All qPCR analyses were conducted with three biological replicates with technical triplicates for each sample. Amplification specificity was verified by melting curve analysis [54].

#### 2.8.3. Field Experiment

The probiotic feed formulation that showed the best physiological performance and vitellogenin expression in the laboratory experiment was selected for field evaluation at the Pantae Community Enterprise, Phatthalung, Thailand, between March and July 2025. Newly divided colonies of *Heterotrigona itama* (2 months after division) with similar initial strength were used in the experiment. A total of 10 colonies were randomly assigned to two dietary treatments (*n* = 5 colonies per treatment): (i) control colonies receiving basal pollen without probiotic supplementation and (ii) probiotic-treated colonies receiving LAB combined with yeast supplementation. The probiotic diet was provided on sterile plastic feeding plates at 20 g per colony and replaced every 7 days throughout the experimental period following the method of Tlak Gajger et al. [55].

##### Feed Consumption

Feed consumption (g/week) was determined by weighing the remaining diet before replacement and calculating the difference between the supplied and residual feed amounts.

##### Colony Growth and Development

Colony weight gain was determined from changes in colony mass recorded during the experimental period. Internal nest structures, including brood cells, pollen pots, and honey pots, were documented at 14-day intervals using standardized photographic imaging. Nest components were quantified from digital images using ImageJ software (https://imagej.net/ij/, version 1.54d; National Institutes of Health, Bethesda, MD, USA) with grid-overlay analysis to estimate the proportional area (mm^2^) occupied by each structure relative to the total nest area. The estimated honey yield (g/colony/year) was calculated from honey pot density and the proportional nest area occupied by honey storage structures.

### 2.9. Effect of Probiotic Supplementation on Gut Microbiota of H. itama

Total genomic DNA was extracted with the QIAamp DNA Stool Mini Kit (Qiagen, Hilden, Germany) following the manufacturer’s instructions. The V3–V4 region of the bacterial 16S rRNA gene was amplified with primers 341F (5′-CCTACGGGNGGCWGCAG-3′) and 805R (5′-GACTACHVGGGTATCTAATCC-3′). Sequencing libraries were prepared and sequenced on the Illumina MiSeq platform using 2 × 300 bp paired-end chemistry. Raw sequencing reads were processed with the QIIME2 pipeline for quality filtering, denoising, chimera removal, and taxonomic classification against the SILVA database. Alpha diversity indices and beta diversity analyses were computed to assess differences in gut microbial communities among treatments. Alpha diversity differences were analyzed using one-way ANOVA or Kruskal–Wallis tests, as appropriate. Microbiome results were interpreted primarily at the compositional level [23,26].

### 2.10. Statistical Analysis

All experiments were conducted using at least three independent biological replicates, and data are presented as mean ± standard deviation (SD). Data normality and homogeneity of variance were assessed using the Shapiro–Wilk and Levene’s tests, respectively. Differences among treatments were analyzed using one-way ANOVA followed by Tukey’s HSD multiple comparison test (*p* < 0.05). Two-way ANOVA was used to assess field-experiment parameters involving treatment and sampling-time effects. Microbiome beta diversity was analyzed using PERMANOVA based on Bray–Curtis dissimilarity matrices. Statistical analyses were performed using IBM SPSS Statistics version 26.

## 3. Results and Discussion

### 3.1. Isolation and Preliminary Screening of LAB from the Gut of H. itama

The gut microbiota of stingless bees plays a crucial role in host nutrition, microbial balance, and overall physiological function. Lactic acid bacteria (LAB) are considered key symbionts for their ability to ferment carbohydrates, produce antimicrobial metabolites, and enhance nutrient availability in fermented pollen or bee bread [1,3,18,19]. In this study, gut-derived bacterial isolates were obtained from *Heterotrigona itama* collected at five geographically distinct locations in Songkhla and Phatthalung provinces. Preliminary screening based on Gram staining, cell morphology, catalase activity, and endospore formation identified 71 isolates, comprising 56 Gram-positive and 15 Gram-negative bacteria. Rod-shaped bacteria predominated, and catalase-negative, non-spore-forming isolates were the most abundant. Based on these phenotypic characteristics, 37 isolates were classified as presumptive LAB (Table S3). Minor variations in bacterial morphology across sampling locations may reflect differences in floral resources and environmental microbial exposure. However, the relatively consistent recovery of presumptive LAB across locations suggests a stable gut-associated microbiota in bees [23,24]. Previous studies have reported diverse LAB communities associated with stingless bees, including *Lactiplantibacillus*, *Weissella*, *Leuconostoc*, *Fructobacillus*, and *Apilactobacillus* species isolated from bee guts, honey, and bee bread [1,56,57]. Nevertheless, studies specifically focusing on gut-derived LAB from *H. itama* remain limited. Therefore, the present findings support the notion that the gut of *H. itama* is a potential reservoir of beneficial host-adapted LAB for probiotic development in stingless bee production systems.

### 3.2. Probiotic Properties of LAB Isolated from the Gut of H. itama

The survival of presumptive LAB isolates under simulated GI conditions is presented in Table 1. Five isolates exhibited relatively high tolerance to simulated gastric (pH 3) and intestinal (pH 7.5) conditions, with low reductions in viable cell counts (≤1–2 log CFU/mL) after 4 h of exposure. All selected isolates maintained viable populations above 10^6^ CFU/mL, indicating robust resistance to acid and bile stress. Initial cell densities ranged from 8.00 to 8.18 log CFU/mL and decreased to 6.28–6.97 log CFU/mL after sequential exposure to simulated GI conditions. Among the tested isolates, BP-2, BP-3, and BPW-B1 showed significantly lower log reductions than BPW-B2 and BPW-B11 (*p* < 0.05), indicating greater tolerance to gastrointestinal stress conditions associated with the stingless bee digestive tract, which generally exhibits mildly acidic conditions in the crop (pH 3–4) and near-neutral conditions in the hindgut [23,34]. Similar tolerance characteristics have been reported for LAB isolated from bee guts, honey, and bee bread, particularly *Apilactobacillus* and *Lactiplantibacillus* species, which are adapted to acidic and osmotic stress in sugar-rich diets within the bee crop [43,44,46]. The observed acid and bile tolerance may be associated with intracellular pH homeostasis, membrane adaptation, and stress-response mechanisms commonly reported in LAB [45,46,47]. Several LAB strains isolated from stingless bee products, including *H. itama*, have demonstrated high tolerance to simulated acid and bile conditions, with survival rates ranging from 70–100% [1,58,59,60]. For instance, *Lactobacillus musae* SGMT17 and *Lactobacillus mindensis* SGMT22 isolated from *H. itama* bee bread, tolerated pH 3 and 0.3% bile salts [59], whereas *Lacticaseibacillus rhamnosus* BBAH2 maintained high viability under pH 2 and 0.3% bile exposure [58]. Cell surface characteristics further supported the probiotic potential of the selected isolates. Hydrophobicity values ranged from 71.42–88.40%, indicating moderate to strong adhesion-related probiotic potential for intestinal epithelial surfaces [22,31,46]. BP-3 and BPW-B1 exhibited significantly higher hydrophobicity (>80%) than BPW-B2 and BPW-B11 (*p* < 0.05), suggesting favorable adhesion-related probiotic properties. Auto-aggregation values ranged from 20.48–35.48%, with only BP-3 and BPW-B1 exceeding the commonly suggested threshold of 30%, indicating stronger microbial clustering and colonization stability [45,61]. Previous studies reported hydrophobicity values of 60–95% and variable auto-aggregation among LAB isolated from bees and bee products. For example, *L. rhamnosus* BBAH2 showed hydrophobicity and auto-aggregation values of 42% and 55%, respectively [58], while LAB isolated from stingless bee bread demonstrated strong auto-aggregation ranging from 57.47–92.77% [59]. In addition, *Bacillus subtilis* HTI-23 isolated from *H. itama* honey exhibited approximately 58% and 61% [60]. Co-aggregation ability was evaluated using *Serratia marcescens*, an opportunistic Gram-negative bacterium linked to gut dysbiosis and increased mortality in honey bees [32]. Co-aggregation with *S. marcescens* ATCC 13880 ranged from 14.86–25.92%, with BP-3 and BPW-B1 showing significantly higher interaction ability than other isolates (*p* < 0.05). This property may contribute to pathogen exclusion by promoting mixed aggregate formation, which limits pathogen attachment to epithelial surfaces (Collado et al., 2010; Lee et al., 2024) [22,61]. Similar co-aggregation levels (10–35%) have been reported in LAB–pathogen interactions [62]. Some LAB strains isolated from stingless bee bread have demonstrated co-aggregation exceeding 30% after 2 h of incubation with *Escherichia coli* and *Salmonella enterica*, indicating strong pathogen-binding ability [58]. These findings indicate strain-dependent differences in adhesion-related properties [31,61]. In the present study, BP-3 and BPW-B1, followed by BP-2, exhibited the strongest tolerance to simulated GI conditions and favorable adhesion-related properties, supporting their potential as host-adapted probiotic candidates for *H. itama*.

### 3.3. Safety Profiling and Coexistence Test

The safety of the selected LAB isolates (BP-2, BP-3, and BPW-B1) was evaluated by assessing hemolytic activity and antibiotic susceptibility (Table S4). All isolates exhibited γ-hemolysis on blood agar, indicating non-pathogenic characteristics and supporting their safety as probiotic candidates, since β-hemolysis is commonly associated with virulence and cytotoxicity [34]. Similar γ-hemolytic profiles have been widely reported among LAB isolated from honey bees and stingless bees, further supporting their biosafety and host compatibility [34,44,47]. Antibiotic susceptibility testing further confirmed the isolates’ safety profile. BP-2, BP-3, and BPW-B1 were sensitive to several clinically important antibiotics, including ampicillin, gentamicin, penicillin G, tetracycline, cephalothin, chloramphenicol, and erythromycin. These findings are consistent with previous studies showing that LAB are generally susceptible to antibiotics that target the cell wall and protein synthesis, suggesting the absence of acquired resistance genes [1,63]. However, all isolates exhibited intrinsic resistance to quinolones (ciprofloxacin, norfloxacin, and nalidixic acid) and sulfonamides, a trait commonly reported in members of the *Lactobacillaceae* and *Leuconostocaceae* families. This natural resistance is primarily associated with structural features of DNA gyrase and limited cellular antibiotic uptake, rather than with acquired resistance genes, and therefore poses minimal risk of horizontal gene transfer [57,64]. Comparable antibiotic susceptibility profiles have also been reported among bee-associated LAB, particularly *Apilactobacillus* and *Lactiplantibacillus* species, which are widely recognized as safe and suitable candidates for probiotic applications [34,44]. Before developing multi-strain probiotics, coexistence among candidate strains must be confirmed to prevent antagonistic interactions that could reduce viability and efficacy [65]. As shown in Figure S1, no visible inhibition zones were observed among BP-2, BP-3, and BPW-B1, indicating good compatibility and the absence of antagonism. Compatible strains are advantageous in multi-strain formulations because they enhance stability, synergistic activity, and host colonization efficiency [10,47]. Therefore, BP-2, BP-3, and BPW-B1 can be effectively applied either as single strains or as a multi-strain probiotic candidate in apiculture.

### 3.4. Molecular and Biochemical Identification of Selected LAB Isolates

**Figure 1:**
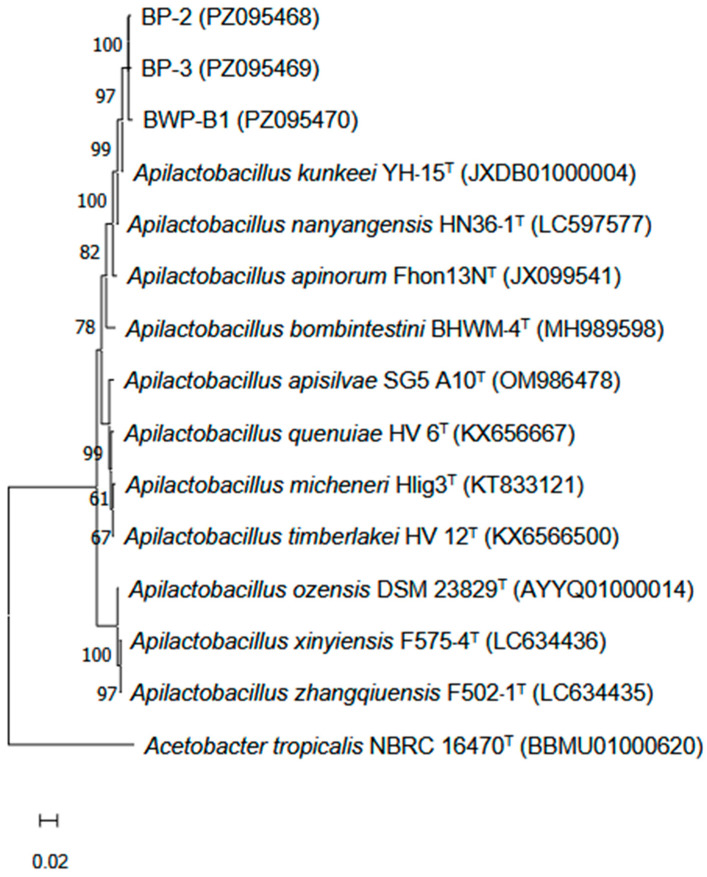
Phylogenetic tree based on partial 16S rRNA gene sequences showing the relationship of LAB isolates (BP-2, BP-3, and BPW-B1) with reference strains. The tree was constructed using the neighbor-joining method, and branch support was evaluated by bootstrap analysis with 1000 replicates. *Acetobacter tropicalis* NBRC 16470^T^ was used as an outgroup. The scale bar represents 0.02 substitutions per nucleotide position.

The selected LAB isolates were identified using molecular and biochemical methods. Phylogenetic analysis of partial 16S rRNA gene sequences showed that BP-2 (accession no. PZ095468), BP-3 (accession no. PZ095469), and BPW-B1 (accession no. PZ095470) clustered within the genus *Apilactobacillus* with high sequence similarity (≥99%), forming a well-supported clade with bee-associated LAB reference strains (Figure 1). All isolates grouped closely with *Apilactobacillus kunkeei* YH-15^T^, indicating strong genetic relatedness, and showed phylogenetic relationships with other bee-associated species, including *A. apinorum*, *A. bombintestini*, and *A. apisilvae*, which are commonly found in sugar-rich niches such as the bee gut, honey, and bee bread [33,66]. In Melipona species, *Apilactobacillus* and *Bombella* dominate the crop, whereas *Apilactobacillus* and other *Lactobacillaceae* members dominate the ventriculus [26,27]. Similar findings have been reported in stingless bees, including the isolation of *Apilactobacillus* spp. from the GI tract of *Melipona beecheii* [57] and the identification of the novel species *Apilactobacillus apisilvae* in *Austroplebeia australis* [67]. Members of the genus *Apilactobacillus*, particularly *A. kunkeei*, are recognized as dominant fructophilic lactic acid bacteria (FLAB) in the bee’s GI tract. These bacteria contribute to disease prevention by producing antimicrobial metabolites (e.g., lactic acid, acetic acid) and other bioactive compounds (e.g., bacteriocins such as kunkeecin), competing with pathogens for intestinal adhesion sites, maintaining gut microbial balance, and stimulating host immune responses. FLAB has shown antagonistic activity against major bee pathogens, including *Ascosphaera apis*, *Nosema ceranae*, *Paenibacillus larvae* [31,33,46]. Their adaptation to fructose-rich and osmotically stressful environments reflects ecological specialization within bee-associated habitats [16,23,29,33]. Biochemical characterization using API 50 CHL (Table S5) and API ZYM (Table S6) further supported the molecular identification. All isolates metabolized major sugars including glucose, fructose, mannitol, sucrose, trehalose, esculin, and potassium gluconate, consistent with adaptation to nectar- and honey-rich environments [29,30]. BPW-B1 also utilized raffinose, indicating minor metabolic variation among isolates. Enzymatic profiling revealed positive activities of leucine arylamidase, acid phosphatase, and naphthol-AS-BI-phosphohydrolase, indicating active protein and phosphate metabolism that supports nutrient turnover in the bee gut. These metabolic traits align with the ecological and probiotic functions of *Apilactobacillus* spp., including nutrient fermentation, organic acid production, pathogen inhibition, and maintenance of gut microbial balance [3,17,43]. Species identification was based primarily on partial 16S rRNA gene sequencing and biochemical characterization; therefore, whole-genome sequencing would provide higher taxonomic resolution among closely related *Apilactobacillus* species.

### 3.5. Effect of LAB–Yeast Fermentation on Physicochemical and Functional Properties of Fermented Bee Bread

Stingless bees rely on pollen and nectar as major nutrient sources. These materials, which undergo microbial fermentation within brood cells, contribute to larval development. Previous studies have shown that bee bread fermentation is mediated by LAB and osmophilic yeasts, particularly *Starmerella* and *Zygosaccharomyces*, which play roles in nutrient metabolism and microbial stability within stingless bee colonies [13,15,40]. In particular, *Zygosaccharomyces* spp. contribute steroid precursors important for larval development [40]. Therefore, selected *A. kunkeei* strains (BP-2, BP-3, and BPW-B1) were co-cultivated with *Z. bailii* TSU_YK2 and *S. meliponinorum* TSU_YP10 for solid-state fermentation of bee pollen to mimic natural bee bread fermentation and to enhance nutritional value.

#### 3.5.1. Protein Content and Free Amino Acids

As expected, solid-state fermentation significantly improved the nutritional quality of bee pollen (Table 2). Inoculation with LAB and yeast co-cultures increased protein content, protein digestibility, titratable acidity, and antioxidant activity compared with the non-inoculated control (*p* < 0.05). Protein content ranged from 29.96 to 41.10 g/100 g, and protein digestibility rose from approximately 52% in the control to over 75% in inoculated treatments. Among the single-strain treatments, *Apilactobacillus kunkeei* BPW-B1 showed the highest digestibility (74.33%). However, the highest protein content (>40 g/100 g) and digestibility (>75%) were observed in FP123, which consisted of mixed LAB strains (BP-2, BP-3, and BPW-B1) combined with the yeasts *Zygosaccharomyces bailii* TSU_YK2 and *Starmerella meliponinorum* TSU_YP10. These findings suggest synergistic interactions between LAB and yeasts that enhanced nutrient availability and protein utilization. The improvement in protein digestibility is primarily due to microbial degradation of the pollen wall structure, particularly the sporopollenin–protein matrix, which normally limits nutrient bioavailability [6,8,10,18]. These results align with previous studies showing that fermentation enhances nutrient release and protein utilization from pollen. For instance, Poyraz et al. (2023) reported a maximum protein digestibility of 61.64 ± 0.018% using mixed cultures of *A. kunkeei* and yeasts [17]. Similarly, fermentation with *A. kunkeei* strains and *Hanseniaspora uvarum* AN8Y27B increased protein digestibility from 62.1% in raw pollen to 75.14% post-fermentation [14]. In addition, microbial consortia containing *Lactobacillus plantarum*, *A. kunkeei*, and *Lactobacillus acidophilus* significantly increased total amino acid content compared with unfermented pollen [18]. Enhanced digestibility may be linked to LAB-derived aminopeptidases, particularly leucine arylamidase, which hydrolyze pollen proteins into free amino acids. Similar improvements after fermentation have been widely reported in legume and pollen substrates, attributed to enzymatic hydrolysis and acid-induced protein denaturation [6,8,9,10,14,31]. The amino acid composition of fermented bee pollen was further analyzed and is presented in Table S7. Mixed fermentation (FP123) significantly increased the levels of most amino acids compared with yeast monoculture and single-strain LAB fermentation, indicating enhanced proteolysis during co-fermentation. Several nutritionally important amino acids, including arginine, proline, leucine, phenylalanine, histidine, glutamic acid, glycine, and serine, were significantly higher in FP123 (*p* < 0.05), whereas the remaining amino acids varied significantly among treatments. Among these, arginine and proline showed the greatest increases compared with the other treatments. The enhanced amino acid profile in FP123 is likely linked to complementary enzymatic activities produced by LAB and yeasts during fermentation. Similar observations have been reported for co-fermentation of *A. kunkeei* and yeasts, with total free amino acids (FAA) increasing significantly from 10.97 to 25.29 g/kg with notable increases in leucine, lysine, alanine, valine, phenylalanine, arginine, tyrosine, and glutamic acid, indicating enhanced proteolysis [14]. Likewise, phenylalanine, alanine, and leucine were reported as the most abundant amino acids in bee pollen fermented by *A. kunkeei*, along with the yeasts *Starmerella magnoliae* and *Zygosaccharomyces siamensis* [17]. In addition, 28 amino acids were identified in bee pollen fermented using a microbial consortium of LAB, with proline detected at the highest concentration [18]. Similarly, FAA levels in co-culture systems increased from 2.12% in unfermented pollen to 2.86–3.20% in monoculture, reaching a maximum of 4.60% [10]. Notably, increased levels of proline and arginine may also contribute to improved nutritional quality, since these amino acids are associated with energy metabolism and physiological functions in bees [4,8,11,33].

#### 3.5.2. Total Acidity and Organic Acid Production

Co-fermentation significantly increased titratable acidity relative to the control (*p* < 0.05) (Table 2). Yeast- and LAB-containing treatments enhanced acidity to 32.18–48.71%, with the highest value observed in FP123 (48.71 ± 1.07%). These results indicate intensified organic acid formation during mixed fermentation. Synergistic interactions between LAB and yeasts likely promote heterofermentative metabolism, thereby increasing the production of short-chain organic acids, particularly lactic and acetic acids, which contribute to pH reduction and improved microbial stability. Similar increases in acidity have been reported in fermented bee pollen and bee bread systems [10,14,17,18,44]. GC–MS chromatograms (Figure S2) showed more prominent organic acid–related peaks in FP123 than in single-strain and yeast-only treatments, indicating enhanced metabolic activity during co-fermentation. The dominant peak at RT 13.89 min was identified as acetic acid, and lactate derivatives, including methyl lactate, ethyl lactate, lactic anhydride, and isoamyl lactate, were also detected. Minor acids, including propanoic, hexanoic, octanoic, and benzoic acids, reflected secondary metabolism of carbohydrates, lipids, and amino acids. Moderate levels of phenolic derivatives further suggested microbial degradation of the pollen matrix and the release of bound phenolic compounds. These metabolites are primarily produced via heterofermentative pathways by LAB, such as *A. kunkeei*, in combination with osmophilic yeasts, contributing to improved biochemical stability. Previous studies reported lactic acid as the predominant metabolite during bee pollen fermentation (0.45–4.40 mg g^−1^), with acetic acid second (up to 2.08 mg g^−1^) [17]. Mixed-culture fermentation also produced high levels of lactic and acetic acids (51–87 g kg^−1^), increased titratable acidity (18.41–25.65 meq NaOH kg^−1^) and lactic acid content (3.46–4.80%) [14,18]. Short-chain fatty acids produced by bee gut symbionts, particularly acetate and propionate, have been linked to energy metabolism, vitellogenin regulation, sucrose sensitivity, and maintenance of microbial stability in bees [28]. These symbionts may modify the intestinal environment by lowering pH and oxygen levels, thereby promoting microbial activity. These findings suggest that LAB–yeast co-fermentation enhances organic acid production and improves the functional properties of fermented pollen.

#### 3.5.3. Antioxidant Activity of Fermented Bee Pollen 

All LAB-inoculated treatments exhibited significantly higher antioxidant activity than the controls (*p* < 0.05), with DPPH radical-scavenging values ranging from 61.72–74.62%. The mixed culture of multi-strain LAB and yeasts (FP123) showed the highest antioxidant activity (Table 2), indicating a synergistic effect of co-fermentation. This enhancement is likely associated with microbial enzymatic activity that disrupts pollen cell walls, releasing bound phenolic compounds and other bioactive constituents. Proteolysis and organic acid production during LAB–yeast fermentation may further enhance the bioavailability of phenolic hydroxyl groups responsible for antioxidant activity [8,9,15,18,44,68]. The increased antioxidant activity observed in this study is consistent with previous reports showing that mixed-culture fermentation enhances phenolic release in bee pollen. Fermented bee pollen has been reported to have higher antioxidant activities than raw pollen. For example, free phenolic content ranging from 12.87 to 15.09 mg GAE g^−1^ has been reported in fermented bee bread [17]. Similarly, fermented pollen showed higher antioxidant activity, with ABTS and DPPH values ranging from 3.71–9.01 and 3.88–7.96 mmol Trolox g^−1^, respectively, and FRAP values ranging from 3.33–5.92 mmol Fe^2+^ g^−1^. These improvements were associated with increased concentrations of phenolic compounds, including quercetin, kaempferol, luteolin, ferulic acid, and p-coumaric acid, which are well-known for their antioxidant properties [11,18,48]. Co-fermentation with multiple LAB strains and yeasts increased radical-scavenging activity from 57.29% to 77.46% (DPPH), 53.16% to 65.87% (hydroxyl radical), and 42.15% to 56.31% (ABTS) after mixed fermentation, indicating synergistic microbial interactions that enhance the transformation of antioxidant compounds [10]. Although phenolic content may vary with floral origin, microbial strains, and fermentation conditions, fermented pollen generally exhibits higher antioxidant activity than unfermented pollen [8,9,10,11,18].

### 3.6. Microbial Dynamics During Bee Pollen Fermentation

Viable cell counts of LAB and yeasts during 14 days of solid-state fermentation are shown in Table 3. All inoculated treatments showed increased microbial populations from Day 0 to Day 7, followed by a slight decline at Day 14, consistent with typical fermentation dynamics [13,15,17,18]. In the yeast monoculture, yeast counts increased from 6.02 to 7.62 log CFU/mL by Day 7, then declined slightly to 7.21 log CFU/mL at Day 14, likely due to nutrient depletion and metabolite accumulation. In LAB-containing treatments, all strains remained viable throughout fermentation, and FP3 and FP123 maintained the highest LAB counts on Day 14 (7.05–7.08 log CFU/mL), indicating high tolerance to acidic fermentation conditions. FP123 also showed stable persistence of both LAB and yeasts, suggesting balanced coexistence and synergistic interactions among microorganisms [14,15,17,33]. Similar microbial dynamics have been widely reported in bee pollen fermentation, with LAB and yeast populations increasing during early fermentation and gradually declining during prolonged storage. For instance, total aerobic bacteria reportedly increased from 5–7 to 9–10 log CFU g^−1^ before declining to 4–6 log CFU g^−1^ during storage. Yeasts increased from 5.0 to 5.2–5.7 log CFU g^−1^ within 3–5 days, then decreased to 2.2–3.6 log CFU g^−1^ after Day 30 [17]. Similarly, LAB populations decreased from approximately 7.0 to 2.0 log CFU g^−1^ during prolonged storage, whereas yeast populations in starter-fermented samples stabilized at 4.0–4.8 log CFU g^−1^ [14]. These results indicate that microbial populations typically peak during the early fermentation stage (Days 5–7) and then gradually decline, reflecting normal fermentation progression and improved microbial stability in starter-culture systems. LAB likely enhance substrate hydrolysis, while yeasts provide vitamins and nitrogen sources that support LAB survival [20]. The maintenance of viable microbial populations above 10^6^–10^7^ CFU/g throughout fermentation further indicates good probiotic stability and suggests that the fermented pollen could serve as an effective functional probiotic feed supplement. Given their stable growth and metabolic compatibility, the FP123 formulation, together with *Z. bailii* TSU_YK2 and *S. meliponinorum* TSU_YP10, was selected for further evaluation as a probiotic supplement for the stingless bee *H. itama* in subsequent cage and field experiments.

### 3.7. Feeding Trial to Evaluate the Performance of Supplemented Diets in H. itama in a Cage Experiment

#### 3.7.1. Physiological Responses of *H. itama* to Probiotic-Supplemented Diets

This study demonstrated synergistic effects of LAB (*A. kunkeei* BP-2, BP-3, and BPW-B1) in combination with stingless bee-associated yeasts (*Z. bailii* TSU_YK2 and *S. meliponinorum* TSU_YP10) on the physiological performance and HPG development of *H. itama*. Significant differences among treatments were observed (*p* < 0.05), indicating that probiotic supplementation was associated with improved feed intake, physiological performance, gland development, and bee survival (Table 4). Average daily intake ranged from 0.054–0.070 g/bee/day, with the highest consumption observed in Diet IV, followed by Diet III and Diet II, compared with the sucrose control (Diet I). The increased intake likely reflects improved palatability and nutrient availability resulting from microbial fermentation. Yeasts such as *Zygosaccharomyces* and *Starmerella* are known to produce enzymes, amino acids, and vitamins that enhance pollen digestibility and nutrient assimilation [41,69]. *Zygosaccharomyces bailii* can survive in harsh conditions such as acidic and high-sugar environments [69]. *Starmerella meliponinorum*, originally isolated from stingless bee (*Tetragonisca angustula*) pollen, is well adapted to the bee environment and contributes to fermentation, nutrient transformation, and biochemical modification of pollen and honey [41]. Body weight gain and abdominal lipid content (ALC) exhibited similar trends, with Diet IV showing significantly higher values (0.045 ± 0.004 g/bee and 262.5 ± 16.8 mg/g, respectively) than the sucrose control (*p* < 0.05). Similar findings have been reported in honey bees (*Apis mellifera*). Probiotic supplementation increased feed consumption to 177.50 ± 26.16 mg/bee, head protein content to 312.62 ± 28.71 µg/mL, and digestibility to 48.41 ± 1.90% [52]. Hasan et al. (2022) also reported an increase in body weight from 118.66 ± 9.57 mg in controls to 124.45–140.90 mg after supplementation with *Bacillus clausii*, *Lactobacillus brevis*, and organic acids [21]. Protein-rich diets further increased lipid reserves by approximately 4–6-fold (0.23–0.29 mg/bee) relative to controls [51]. Previous studies reported fat body levels ranging from 0.11 to 0.90 mg/bee depending on diet quality and environmental conditions [7], highlighting the importance of lipid reserves for bee energy balance and physiological fitness. HPG development was evaluated using acini size and protein content, both important indicators of nurse bee function and brood food production capacity in stingless bees [2]. HPG development, assessed by acini size and protein content, was also significantly enhanced in Diet IV. Bees fed Diet IV had larger acini (2648.9 ± 44.3 µm^2^) than those fed the sucrose control diet (2315.6 ± 42.8 µm^2^), together with the highest HPG protein content (30.4 ± 2.5 mg/g tissue) (*p* < 0.05), suggesting enhanced gland development and secretory activity (Figure S3). Previous studies also reported that probiotic supplementation increased HPG size, protein synthesis, and royal jelly production in honey bees. For example, Hassan and Elenany (2024) reported that probiotic-enriched soybean patties improved HPG development in *A. mellifera* nurse bees, resulting in larger gland diameter (14.89 ± 0.097 µm), greater surface area (0.065 ± 0.001 µm^2^), and higher royal jelly production of up to 219 mg compared with the control diet [36]. Commercial EM^®^ probiotics containing LAB, yeast, and photosynthetic bacteria increased HPG size in nurse bees from 26–28 µm in controls to 44–46 µm, a 60–70% increase by day 11 [55]. Similarly, *Lactobacillus brevis* supplementation increased HPG size by 17–50%, reaching 0.027 ± 0.007 mm^2^ on Day 6, while enhancing protein synthesis by up to 47% and digestibility by 3–9% [70]. Enhanced HPG development may result from increased availability of essential amino acids, vitamins, and microbial metabolites that support protein metabolism and gland maturation. HPG secretions contain digestive enzymes essential for larval nutrition, and higher protein content indicates enhanced biosynthetic activity, particularly during the nurse bee stage (13–17 days post-emergence) [2,49,70]. Survival performance also differed significantly among treatments. After 14 days, Diet IV showed the highest survival rate (92.6 ± 2.7%), followed by Diet III (86.4 ± 3.2%) and Diet II (80.2 ± 3.9%), whereas the sucrose-only diet showed the lowest survival. These results indicate the nutritional limitations of carbohydrate-only diets, which lack essential amino acids, lipids, and micronutrients necessary to maintain physiological homeostasis. Similar improvements in longevity and brood survival have been reported among probiotic-supplemented honey bees. Probiotic supplementation at 2.5–10% improved adult bee survival, maintaining approximately 78% survival in EM-treated groups by day 30 without significantly altering feed consumption [55]. In addition, *Lactobacillus brevis* supplementation increased bee lifespan to 26.04 days, about 2–3 days longer than the syrup-only control [70]. Similarly, Brewer’s yeast supplementation improved survival to 29.00 ± 2.83 days in *A. mellifera* [52] while a probiotic mixture containing *Bifidobacterium bifidum*, *B. longum*, *B. infantis*, *Lactobacillus casei*, *L. acidophilus*, and *L. lactis* further increased brood survival by up to 95% within 18 days and increased pollen collection to 621.67 g compared with 458.67 g in control colonies [71]. These findings support the role of multi-strain probiotics in enhancing physiological performance, gland development, and survival in *H. itama*.

#### 3.7.2. Vitellogenin Expression

**Figure 2:**
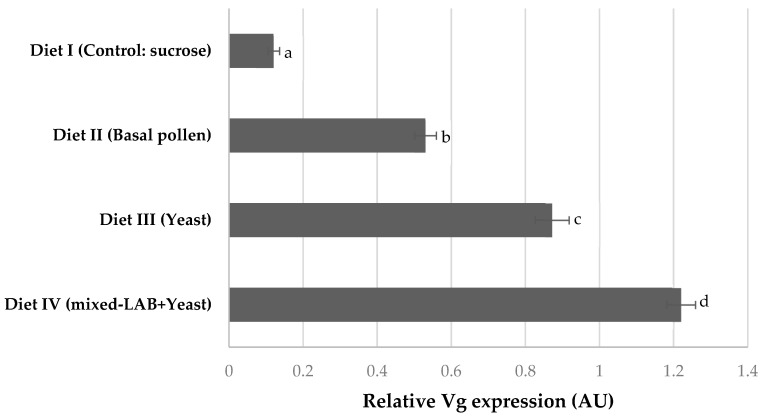
Relative expression of vitellogenin (Vg) in *Heterotrigona itama* workers fed different dietary treatments. Expression levels were normalized to β-actin and expressed as arbitrary units (AU). Values are mean ± SD (*n* = 5 biological replicates). Different letters indicate significant differences (one-way ANOVA, Tukey’s HSD, *p* < 0.05).

The relative expression of vitellogenin (Vg) in *H. itama* workers fed different diets is shown in Figure 2. Bees fed the LAB–yeast supplemented diet (Diet IV) showed the highest Vg expression, followed by Diet III, Diet II, and the sucrose control (Diet I) (*p* < 0.05), indicating improved nutritional and physiological status after probiotic supplementation. The synergistic effects of LAB and yeast likely enhanced nutrient bioavailability and the metabolic activity involved in Vg synthesis [38,39,55]. Similar findings have been reported previously. Güneşdoğdu et al. (2024) observed stable Vg levels of 4.66–4.73 ng/mL in probiotic-treated bees, suggesting stable physiological status across probiotic treatments and balanced nutrient availability in worker bees [51] while Garrido et al. (2024) reported higher Vg expression in bees supplemented with *A. kunkeei* (about 2.3 AU) and *Bifidobacterium coryneforme* (about 2.2 AU), approximately 2.2–2.3-fold higher than controls, and a moderate increase was detected in bees treated with *L. plantarum* (~1.7 AU) [54]. In addition, feeding bees fermented broth of the probiotic *Leuconostoc mesenteroides* TBE-8 significantly enhanced expression of immunity-related genes, including MRJP1 (1400-fold) and vitellogenin (20-fold), and increased expression of antimicrobial peptides hymenoptaecin (17-fold) and apidaecin (7-fold) [72]. Vitellogenin (Vg) is a glycolipoprotein synthesized in the fat body and plays important roles in nutrient storage, immunity, longevity, and physiological regulation in bees. It is commonly used as an indicator of nutritional status and dietary quality. Head protein levels are closely associated with HPG development, which is essential for royal jelly production and larval nutrition. In addition, diet strongly influences the composition of the gut microbiota, thereby affecting digestion efficiency, immune responses, and overall bee health [2,33,38,39,51,52,54]. In this study, higher relative Vg expression in probiotic-fed bees was associated with HPG development, larger acini, higher protein content, and greater survival. Diet IV, which contained multiple LAB strains (*A. kunkeei* BP-2, BP-3, and BPW-B1) along with *Z. bailii* TSU_YK2 and *S. meliponinorum* TSU_YP10, produced the highest HPG protein levels and survival rate, indicating improved physiological stability and nutritional balance in *H. itama* workers.

### 3.8. Field Feeding Trial of Probiotic-Supplemented Diet in H. itama

From February to July 2025, the optimized probiotic feed containing multi-strain LAB and yeasts was applied to newly divided *H. itama* colonies at the Pantae community enterprise in Phatthalung, Thailand, to evaluate its effectiveness under field conditions. Probiotic-treated colonies developed larger, more organized brood structures and higher densities of pollen and honey pots than control colonies (Figure S4), indicating improved colony establishment and resource storage capacity. Feed intake increased progressively in both groups during the 5-month trial; however, probiotic-treated colonies consistently consumed significantly more feed (13.6–17.2 g/week) than controls (8.9–13.1 g/week) (*p* < 0.05) (Table 5). Increased feed intake was associated with higher egg cell production (11.6–12.4 cells vs. 8.2–8.9 cells in controls) and significantly larger brood areas with probiotic colonies reaching approximately 446,688 mm^2^ compared with 175,620 mm^2^ in controls. Colony biomass also increased significantly, with probiotic colonies reaching up to 0.31 kg, compared with 0.20 kg in controls (*p* < 0.05). Notably, honey production was consistently higher in probiotic-treated colonies, with estimated yields of 520–555 g/colony/year, whereas control colonies produced only 221–273 g/colony/year during the same period. A temporary decline in honey yield was observed in month 4 despite large brood areas, suggesting that nutrients were preferentially allocated to brood development and colony expansion rather than to honey storage. Honey production increased again in month 5 as colony structure stabilized and worker populations grew, indicating improved foraging efficiency and nectar storage (Figure S4). Significant positive correlations among egg cell number, brood area, colony weight gain, and honey yield further underscored the role of improved nutritional status in supporting reproductive performance, colony development, and overall productivity. These findings align with previous studies showing that microbial supplementation improves colony strength and productivity in social bees. Kafle and Huang (2025) reported that commercial probiotics (Tensall Bio-Tech Co., Ltd., Yilan County, Taiwan) containing *Bifidobacterium* spp. and *Lactobacillus* spp. increased honey production in European *A. mellifera* by approximately 25–34%, reaching 22.28 ± 0.44 kg/hive, compared with 16.73 ± 0.73 kg/hive in control colonies under field conditions [71]. Similar findings were reported by García-Vicente et al. (2023), who observed increases in adult bee populations from 6258 to 8295 and in brood area from 8.16 to 21.46 dm^2^ following probiotic supplementation [73]. Likewise, Alberoni et al. (2018) reported improvements in brood population (46.2%), pollen stores (53.4%), and honey yield (59.2%) [35]. These results confirm that supplementation with multi-strain LAB and yeast can enhance colony development, structural stability, and honey production in *H. itama*.

### 3.9. Impact of Probiotic Supplementation on Gut Microbial Composition in H. itama

**Figure 3:**
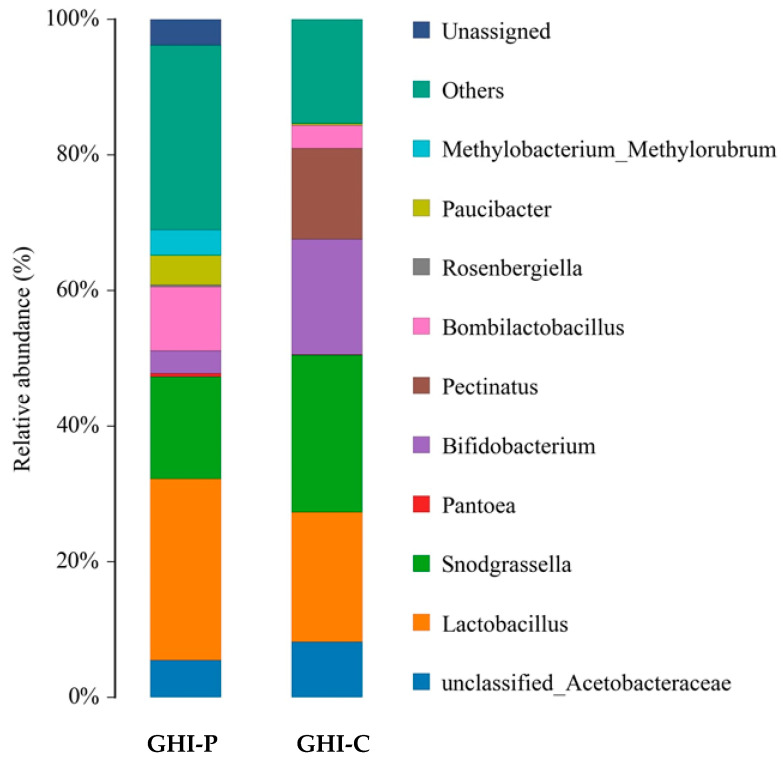
Relative abundance (%) of dominant bacterial genera in probiotic-treated (GHI-P) and untreated control (GHI-C) groups. Probiotic supplementation significantly enriched bacterial taxa commonly associated with the core bee microbiota, particularly *Lactobacillus*, *Snodgrassella*, and *Bifidobacterium*, while reducing the prevalence of unassigned and low-abundance taxa. These shifts suggest changes toward bacterial taxa commonly associated with the core bee microbiota. Significant differences in microbial composition among treatments were confirmed by PERMANOVA (*p* < 0.05).

Honey bees have a host-specific core gut microbiota, primarily composed of *Snodgrassella*, *Gilliamella*, *Lactobacillus*, *Bombilactobacillus*, and *Bifidobacterium*, which together account for 95–99% of the colony’s bacterial population. These microbes support digestion, nutrient metabolism, and immune function and may help protect against pathogens. Although the gut microbiota is generally conserved across bee species, including stingless bees, its composition can vary with diet, environment, developmental stage, antibiotics, and other stressors, highlighting the importance of maintaining microbial balance for optimal bee health and colony performance [12,74]. Figure 3 illustrates the relative abundance profiles, revealing a clear shift in gut microbiota composition after probiotic supplementation. The treated group exhibited enrichment of key beneficial taxa, particularly *Lactobacillus*, *Bifidobacterium*, and *Snodgrassella*, which are recognized as core symbionts. This shift toward core symbiont dominance suggests increased specialization of gut microbiota in carbohydrate metabolism, organic acid production, and pathogen inhibition, thereby improving host physiological performance [23,24,26].

In parallel, the probiotic group showed fewer unassigned and non-core taxa, indicating a more structured and functionally specialized microbial community, whereas the control group exhibited a more heterogeneous profile. Although *Bombilactobacillus* and other LAB were detected in both groups, their higher abundance in the probiotic treatment emphasizes the role of supplementation in strengthening beneficial microbial networks. Community differences were significant according to PERMANOVA (*p* < 0.05). The probiotic group also showed greater richness (ACE = 4390.37; Chao1 = 3238.95) and diversity (Shannon = 5.72), Good’s coverage (>0.98) confirmed sufficient sequencing depth for robust microbiome characterization. The results are consistent with previous studies showing that beneficial gut bacteria, particularly LAB and *Bifidobacteria*, play important roles in intestinal health, pathogen inhibition, and immune modulation in bees and other insects [26,33,54,74]. Importantly, microbiome modulation was associated with increased vitellogenin (Vg) expression, likely reflecting improved nutritional status and metabolic efficiency in for bees fed probiotics [4,19,51,70,75]. Similarly, Kim et al. (2024) [52] reported that probiotic diets significantly altered the bee gut microbiota, with *Lactobacillus* as the dominant genus (34–91% relative abundance), followed by *Bifidobacterium*, *Snodgrassella*, and *Gilliamella*. Supplementation with *Apilactobacillus* and *Lactiplantibacillus* also increased microbial richness and the abundance of core symbionts, including *Frischella*, *Snodgrassella*, *Gilliamella*, *Bombilactobacillus*, and *Commensalibacter* [70]. These microbes contribute to carbohydrate digestion, regulation of vitellogenin expression, improved body weight and gut development, and suppression of potential pathogens [5]. In stingless bees (*Melipona* spp.), gut bacteria also exhibit spatial organization, with *Apilactobacillus* and *Bombella* predominating in the crop, *Lactobacillaceae* in the midgut, and *Bifidobacterium* and *Lactobacillus* dominating the rectum. Despite taxonomic differences among eusocial bees, the stingless bee gut microbiota exhibit conserved roles in nutrient metabolism, digestion, and host health [25,26,27]. Emerging evidence suggests that host-adapted probiotics, particularly LAB consortia, are more effective than non-native probiotics (e.g., food-derived *Lactobacillus* and *Bifidobacterium*) because they colonize the bee gut more effectively, stimulate immune and metabolic pathways (e.g., defensin, abaecin, vitellogenin, and insulin signaling), and suppress pathogens such as *Paenibacillus larvae*, *Nosema*, and *Serratia* [26,37,55,74]. However, microbiome results in the present study were interpreted primarily at the compositional level, whereas the microbiota’s functional metabolic activity could not be directly determined. Future probiotic development should prioritize host-specific microbial consortia, standardized formulations, and colony-level evaluation to ensure long-term benefits for bee health and productivity. Stingless bee nests harbor diverse bacteria, yeasts, and fungi that support food fermentation, nutrient transformation, and pathogen protection. Key bacterial genera such as *Lactobacillus*, *Bacillus*, and *Fructobacillus* produce enzymes and antimicrobial compounds that enhance pollen and nectar utilization, and inhibit pathogens such as *Paenibacillus larvae*, while yeasts, including *Starmerella* and *Zygosaccharomyces*, ferment sugars and support larval development through nutritional and hormonal interactions [12,15,26,40,75]. These findings highlight a coordinated microbiome–host interaction in which probiotic-driven microbial modulation enhances metabolic efficiency, immune-related gene expression, colony health, and productivity. To the best of our knowledge, few studies have investigated probiotic supplementation in stingless bees, particularly integrating both laboratory and field validation. The present study provides comprehensive evidence that supplementation with multi-strain LAB and yeast improves physiological performance, colony health, and colony productivity in *H. itama*, supporting the potential application of functional probiotics for sustainable stingless bee management.

## 4. Conclusions

This study developed a host-adapted probiotic system for stingless bees using gut-derived lactic acid bacteria (LAB) and osmophilic yeasts to enhance bee pollen fermentation and colony health. Three strains identified as *Apilactobacillus kunkeei* (BP-2, BP-3, and BPW-B1) demonstrated strong probiotic potential and were confirmed to be biosafety compliant. LAB–yeast co-fermentation significantly enhanced the nutritional and functional properties of pollen, including protein digestibility, titratable acidity, and antioxidant activity, with the multi-strain consortium producing the greatest effects. Laboratory and field evaluations demonstrated improved physiological performance, brood development, honey yield, beneficial core gut microbiota, and overall colony health. This finding provides the first evidence that host-adapted probiotic fermentation can improve feed quality and biological performance in stingless bees, supporting its potential for sustainable apiculture. The proposed probiotic fermentation strategy may also offer practical benefits for stingless bee management, particularly during periods of nutritional stress and limited floral availability.

## Figures and Tables

**Table 1:** Survival and adhesion-related properties of presumptive LAB isolates under simulated gastrointestinal stress conditions.

Isolate	Acid Tolerance (pH 3) log CFU/mL		Acid log Reduction	Bile Tolerance (pH 7.5) log CFU/mL		Bile log Reduction	Hydro- Phobicity (%)	Auto-Aggregation (%)	Co-Aggregation (%)
	0 h	4 h		0 h	4 h				
BP-2	8.04 ± 0.07 ^a^	6.92 ± 0.09 ^ab^	1.12 ± 0.08 ^a^	8.10 ± 0.06 ^a^	7.10 ± 0.08 ^a^	1.00 ± 0.07 ^a^	82.19 ± 1.24 ^b^	24.86 ± 1.21 ^b^	15.34 ± 0.92 ^b^
BP-3	8.18 ± 0.09 ^a^	6.92 ± 0.10 ^ab^	1.26 ± 0.09 ^a^	8.00 ± 0.08 ^a^	6.93 ± 0.09 ^a^	1.07 ± 0.08 ^a^	88.40 ± 1.08 ^a^	35.48 ± 1.39 ^a^	17.88 ± 0.98 ^a^
BPW-B1	8.18 ± 0.08 ^a^	6.97 ± 0.11 ^a^	1.21 ± 0.10 ^a^	8.16 ± 0.07 ^a^	6.91 ± 0.10 ^a^	1.25 ± 0.09 ^a^	87.75 ± 1.15 ^a^	33.72 ± 1.34 ^a^	17.12 ± 0.94 ^a^
BPW-B2	8.07 ± 0.06 ^a^	6.34 ± 0.12 ^bc^	1.73 ± 0.11 ^b^	8.11 ± 0.08 ^a^	6.39 ± 0.11 ^bc^	1.72 ± 0.10 ^b^	73.85 ± 1.48 ^c^	21.36 ± 1.04 ^c^	12.68 ± 0.82 ^c^
BPW-B11	8.05 ± 0.08 ^a^	6.28 ± 0.11 ^c^	1.77 ± 0.10 ^b^	8.02 ± 0.07 ^a^	6.35 ± 0.10 ^c^	1.67 ± 0.09 ^b^	71.42 ± 1.63 ^c^	20.48 ± 0.98 ^c^	11.54 ± 0.80 ^c^

Values represent mean ± SD (*n* = 3). Initial (0 h) and post-exposure (4 h) viable counts are presented to demonstrate survival dynamics under simulated gastrointestinal conditions and to allow direct calculation of log reduction values. Hydrophobicity was determined using the BATH method. Auto-aggregation and co-aggregation were measured after 4 h incubation at 35 ± 2 °C. Co-aggregation was evaluated against *Serratia marcescens* ATCC 13880. Different superscript letters within the same column indicate significant differences (*p* < 0.05) according to one-way ANOVA followed by Tukey’s HSD test.

**Table 2:** Effects of LAB–yeast fermentation on protein content, protein digestibility, titratable acidity, and antioxidant activity of fermented bee pollen after 14 days.

Treatment	Protein (g/100 g)	Protein Digestibility (%)	Titratable Acidity * (%)	DPPH Scavenging (%)
Control (C)	29.96 ± 0.25 ^c^	52.33 ± 1.25 ^e^	12.84 ± 0.72 ^e^	38.15 ± 1.84 ^e^
Control (CY)	31.10 ± 0.32 ^bc^	58.67 ± 1.02 ^d^	18.42 ± 0.95 ^d^	45.28 ± 1.66 ^d^
FP1	39.28 ± 0.41 ^ab^	72.67 ± 1.15 ^b^	32.18 ± 1.12 ^c^	63.84 ± 2.10 ^c^
FP2	37.94 ± 0.38 ^b^	69.67 ± 1.08 ^c^	34.05 ± 0.98 ^c^	61.72 ± 1.94 ^c^
FP3	40.22 ± 0.44 ^a^	74.33 ± 1.42 ^ab^	33.26 ± 1.05 ^c^	66.55 ± 2.08 ^b^
FP12	38.65 ± 0.36 ^ab^	67.67 ± 1.34 ^c^	41.72 ± 1.20 ^b^	64.82 ± 2.26 ^bc^
FP13	39.74 ± 0.47 ^a^	68.33 ± 1.27 ^c^	43.10 ± 1.16 ^b^	67.94 ± 2.41 ^b^
FP23	39.11 ± 0.42 ^ab^	71.00 ± 1.19 ^bc^	44.32 ± 1.08 ^b^	69.88 ± 2.36 ^b^
FP123	41.10 ± 0.42 ^a^	75.67 ± 1.21 ^a^	48.71 ± 1.07 ^a^	74.62 ± 2.85 ^a^

Values are expressed as mean ± standard deviation (*n* = 3). Different superscript lowercase letters (a–e) within the same column indicate significant differences among treatments (*p* < 0.05), as determined by one-way ANOVA followed by Tukey’s HSD test. * Titratable acidity is expressed as % lactic acid equivalent. Protein values are on a dry weight basis. DPPH scavenging activity is expressed as % inhibition.

**Table 3:** Viable counts of LAB and yeasts during 14-day bee pollen fermentation.

Starter Culture Formulation	Microorganism	Viable Counts (log CFU/g)		
		Day 0	Day 7	Day 14
Control (C)	Yeast	ND	ND	ND
	LAB	ND	ND	ND
Control yeast (CY)	Yeast	6.02 ± 0.05 ^c^	7.62 ± 0.07 ^b^	7.21 ± 0.09 ^b^
	LAB	ND	ND	ND
FP1 (BP-2)	Yeast	6.01 ± 0.04 ^c^	7.21 ± 0.06 ^b^	6.62 ± 0.08 ^c^
	LAB	7.05 ± 0.05 ^c^	7.68 ± 0.04 ^ab^	6.73 ± 0.07 ^c^
FP2 (BP-3)	Yeast	6.00 ± 0.05 ^c^	7.08 ± 0.07 ^c^	6.51 ± 0.06 ^c^
	LAB	7.03 ± 0.04 ^c^	7.55 ± 0.05 ^b^	6.66 ± 0.06 ^c^
FP3 (BPW-B1)	Yeast	6.03 ± 0.03 ^c^	8.02 ± 0.06 ^a^	7.35 ± 0.08 ^a^
	LAB	7.06 ± 0.06 ^c^	7.74 ± 0.04 ^a^	7.08 ± 0.05 ^a^
FP12 (BP-2 + BP-3)	Yeast	6.00 ± 0.03 ^c^	7.05 ± 0.07 ^c^	6.40 ± 0.09 ^c^
	LAB	7.04 ± 0.04 ^c^	7.48 ± 0.06 ^b^	6.18 ± 0.08 ^d^
FP13 (BP-2 + BPW-B1)	Yeast	6.02 ± 0.04 ^c^	7.46 ± 0.06 ^b^	6.79 ± 0.07 ^c^
	LAB	7.05 ± 0.05 ^c^	7.63 ± 0.05 ^ab^	6.42 ± 0.07 ^cd^
FP23 (BP-3 + BPW-B1)	Yeast	6.01 ± 0.05 ^c^	7.58 ± 0.07 ^b^	6.94 ± 0.06 ^bc^
	LAB	7.03 ± 0.04 ^c^	7.66 ± 0.05 ^ab^	6.71 ± 0.06 ^c^
FP123 (BP-2 + BP-3 + BPW-B1)	Yeast	6.04 ± 0.03 ^c^	7.72 ± 0.08 ^ab^	7.18 ± 0.07 ^b^
	LAB	7.06 ± 0.06 ^c^	7.79 ± 0.04 ^a^	7.05 ± 0.05 ^a^

Values are expressed as mean ± standard deviation (*n* = 3). Different superscript lowercase letters (a–d) within the same column indicate significant differences among treatments at each sampling time (*p* < 0.05), as determined by one-way ANOVA followed by Tukey’s HSD test. ND = not detected. Viable populations of LAB and yeasts remained above 10^6^–10^7^ CFU/g during fermentation, indicating acceptable probiotic stability under solid-state fermentation conditions.

**Table 4:** Effects of supplemented diets on physiological traits, hypopharyngeal gland development, and survival performance of *H. itama*.

Parameter	Diet I (Control)	Diet II (Basal Pollen)	Diet III (Yeasts)	Diet IV (FP123 + Yeasts)
Physiological traits				
Average daily intake (g/bee/day)	0.054 ± 0.003 ^c^	0.059 ± 0.003 ^bc^	0.063 ± 0.003 ^b^	0.070 ± 0.004 ^a^
Body weight change (g/bee)	0.018 ± 0.003 ^c^	0.025 ± 0.003 ^bc^	0.033 ± 0.004 ^b^	0.045 ± 0.004 ^a^
Abdominal lipid content (mg/g tissue)	161.3 ± 12.4 ^c^	193.8 ± 13.1 ^b^	221.7 ± 14.6 ^b^	262.5 ± 16.8 ^a^
Hypopharyngeal gland development				
Average acini size (µm^2^)	2315.6 ± 42.8 ^c^	2479.3 ± 38.6 ^b^	2518.7 ± 40.1 ^b^	2648.9 ± 44.3 ^a^
Protein content (mg/g tissue)	19.2 ± 1.9 ^c^	24.1 ± 2.0 ^b^	26.3 ± 2.3 ^b^	30.4 ± 2.5 ^a^
Survival				
Survival rate (%)	72.0 ± 4.5 ^c^	80.2 ± 3.9 ^bc^	86.4 ± 3.2 ^b^	92.6 ± 2.7 ^a^

Values are mean ± SD (*n* = 5 cages per treatment, 25 bees per cage). Different superscript letters within rows indicate significant differences among treatments (one-way ANOVA followed by Tukey’s HSD test, *p* < 0.05). Diet I: 50% sucrose (control); Diet II: basal pollen; Diet III: yeasts (*Z. bailii* TSU_YK2 and *S. meliponinorum* TSU_YP10); Diet IV: multi-strain probiotic (FP123 LAB + yeasts).

**Table 5:** Effects of probiotic supplementation on feed intake, colony development, and estimated honey yield of *H. itama* during a 6-month field experiment.

Month	Treatment	Diet Consumed (g/Week)	Egg Cell Count (Cells)	Colony Weight Gain (kg)	Brood Area (mm^2^)	Estimated Honey Yield (g/Colony/Year)
0	Control	8.9 ± 1.3 ^d^	5.4 ± 1.0 ^d^	0.10 ± 0.02 ^d^	60,450 ± 4880 ^e^	221 ^e^
	Probiotic	10.8 ± 1.5 ^cd^	6.2 ± 1.1 ^cd^	0.12 ± 0.03 ^cd^	70,200 ± 5200 ^de^	360 ^c^
1	Control	10.2 ± 1.6 ^d^	6.8 ± 1.3 ^cd^	0.14 ± 0.03 ^cd^	95,880 ± 6750 ^d^	240 ^de^
	Probiotic	13.6 ± 1.9 ^bc^	8.4 ± 1.6 ^bc^	0.18 ± 0.04 ^bc^	117,000 ± 8430 ^cd^	420 ^b^
2	Control	12.4 ± 1.8 ^c^	8.2 ± 1.5 ^bc^	0.19 ± 0.04 ^bc^	162,540 ± 11,220 ^c^	260 ^d^
	Probiotic	17.2 ± 2.3 ^a^	11.6 ± 2.1 ^a^	0.27 ± 0.05 ^a^	216,450 ± 15,220 ^b^	555 ^a^
3	Control	13.1 ± 1.7 ^bc^	8.9 ± 1.6 ^bc^	0.21 ± 0.04 ^b^	185,330 ± 13,440 ^bc^	273 ^d^
	Probiotic	15.9 ± 2.1 ^ab^	10.3 ± 1.8 ^ab^	0.24 ± 0.04 ^ab^	269,100 ± 18,450 ^b^	460 ^b^
4	Control	11.3 ± 1.5 ^cd^	7.1 ± 1.3 ^cd^	0.16 ± 0.03 ^c^	150,420 ± 10,880 ^c^	234 ^de^
	Probiotic	12.7 ± 1.8 ^c^	8.1 ± 1.5 ^bc^	0.19 ± 0.03 ^bc^	362,700 ± 20,310 ^ab^	330 ^c^
5	Control	12.6 ± 1.7 ^c^	8.5 ± 1.6 ^bc^	0.20 ± 0.04 ^bc^	175,620 ± 12,540 ^c^	266 ^d^
	Probiotic	16.8 ± 2.2 ^a^	12.4 ± 2.0 ^a^	0.31 ± 0.05 ^a^	446,688 ± 25,740 ^a^	520 ^a^

Values are mean ± SD (*n* = 5 colonies per treatment). Different superscript letters within columns indicate significant differences among treatments and sampling months (two-way ANOVA followed by Tukey’s HSD test, *p* < 0.05). Feed intake was recorded weekly. Egg cell number, brood area, and colony structure were quantified using photographic analysis with ImageJ. Colony weight gain was calculated from changes in hive mass. Estimated honey yield (g/colony/year) was derived from honey pot density and proportional nest area. Control = basal pollen diet; Probiotic = diet containing multi-strain LAB and yeasts.

## Data Availability

The original data presented in the study are openly available in NCBI GenBank database at PZ095468, PZ095469 and PZ095470.

## Supplementary Materials

The following supporting information can be downloaded at: https://www.mdpi.com/article/10.3390/microorganisms14071415/s1, Table S1: Geographic locations and environmental classification of stingless bee *H. itama* sampling sites in southern Thailand; Table S2: Experimental treatments of LAB–yeast co-culture starter formulations used for bee pollen fermentation; Table S3: Preliminary screening of presumptive LAB isolates from gut-derived bacteria of *H. itama* collected from different geographical locations; Table S4: Safety assessment of LAB isolates based on hemolytic activity and antibiotic susceptibility; Table S5: Carbohydrate utilization profile of LAB isolates determined by API 50 CHL system; Table S6: Enzymatic profiles of LAB isolates determined using API ZYM system; Table S7: Amino acid profile of bee pollen fermented by osmophilic yeasts and probiotic LAB in single- and mixed-cultures; Figure S1: Compatibility matrix of LAB isolates based on co-cultivation assay; Figure S2: GC-MS chromatogram of distinct metabolite profiles in fermented bee pollen; Figure S3: Hypopharyngeal glands of *Heterotrigona itama* workers after 7 days of feeding; Figure S4: Comparison of the internal nest architecture of *H. itama* colonies under different dietary treatments over a 6-month period.
