# Supplementary material for: Host-Adapted Apilactobacillus kunkeei and Yeast Co-Fermentation Improves Fermented Bee Pollen Quality and Physiological Performance in Heterotrigona itama
Source: Microorganisms. 2026 Jun 28;14(7):1415. doi: 10.3390/microorganisms14071415 (PMC13413786; doi:10.3390/microorganisms14071415)
Supplement: Supplementary file 1 [file microorganisms-14-01415-s001.zip › microorganisms-4318575-supplementary.pdf]

Supplementary Material (Table)

Table S1 Geographic locations and environmental classification of stingless bee *Heterotrigona itama* sampling sites in southern Thailand

Site code	Location name	District	Province	Latitude (N)	Longitude (E)	Environment classification
S1	Ko Lae Nang farm	Thepha	Songkhla	6.8219	100.9586	Agricultural–rural
S2	Nada Farm	Rattaphum	Songkhla	7.0386	100.2474	Agricultural
S3	Yingyuan Apiary	Bang Klam	Songkhla	7.0235	100.4662	Semi-urban
S4	Pan Tae	Khuan Khanun	Phatthalung	7.6179	100.0925	Agricultural–forest edge
S5	Rak Phueng Khao Banthat	Tamot	Phatthalung	7.3628	99.9315	Tropical forest

Table S2 Experimental treatments of LAB–yeast co-culture starter formulations used for bee pollen fermentation

<table>
<tr><th>Designated code</th><th>Treatment</th><th>LAB[a]</th><th>Yeast[b]</th></tr>
<tr><td>Control C</td><td>no microorganisms</td><td>- LAB</td><td>- yeast</td></tr>
<tr><td>Control CY</td><td>yeast</td><td>- LAB</td><td>Z. bailii TSU_YK2
+ S. meliponinorum TSU_YP10</td></tr>
<tr><td>FP1</td><td rowspan="3">Single strain</td><td>LAB 1</td><td rowspan="7">Z. bailii TSU_YK2
+ S. meliponinorum TSU_YP10</td></tr>
<tr><td>FP2</td><td>LAB 2</td></tr>
<tr><td>FP3</td><td>LAB 3</td></tr>
<tr><td>FP12</td><td rowspan="4">Multiple strains</td><td>LAB 1 + LAB 2</td></tr>
<tr><td>FP13</td><td>LAB 1 + LAB 3</td></tr>
<tr><td>FP23</td><td>LAB 2 + LAB 3</td></tr>
<tr><td>FP123</td><td>LAB 1 + LAB 2 + LAB 3</td></tr>
</table>

[a]LAB 1, LAB 2, and LAB 3 represent selected LAB isolates evaluated as probiotic candidates. [b]Yeast strains consisted of *Zygosaccharomyces bailii* TSU_YK2 and *Starmerella meliponinorum* TSU_YP10 isolated from bee bread of *H. itama*. Control (C) contained no microorganisms, while Control (CY) contained only yeast strains. Single-strain and multi-strain LAB treatments were combined with yeast strains to evaluate microbial compatibility and co-cultivation potential.

Table S3 Preliminary screening of presumptive LAB isolates from gut-derived bacteria of *Heterotrigona itama* collected from different geographical locations

**Geographical location**	**No. of selected isolates**	**Gram staining**		**Cell morphology**		**Catalase test**		**Endospore formation**	
		Gram +	Gram −	Bacilli	Cocci	+	−	+	−
Ko Lae Nang Songkhla	16	13	3	10	6	7	9	5	11
Nada Farm, Songkhla	14	11	3	9	5	6	8	4	10
Yingyuan Apiary, Songkhla	13	10	3	8	5	5	8	4	9
Pan Tae, Phatthalung	15	12	3	10	5	7	8	5	10
Rak Phueng Khao Banthat, Phatthalung	13	10	3	8	5	5	8	4	9
**Total**	**71**	**56**	**15**	**45**	**26**	**30**	**41**	**22**	**49**

Table S4 Safety assessment of LAB isolates based on hemolytic activity and antibiotic susceptibility

**Isolate**	**Hemo lysis**	**Antibiotic susceptibility profile of LAB isolates**													
		**AMP**	**HLG**	**CIP**	**NOR**	STR	**PEN**	TET	**KF**	NA	**SXT**	**CPD**	CHL	VAN	ERY
**BP-2**	γ	S	S	R	R	S	S	S	S	R	R	I	S	R	S
**BP-3**	γ	S	S	R	R	S	S	S	S	R	R	R	S	R	S
**BPW-B1**	γ	S	I	R	R	S	S	S	S	R	R	I	S	R	S

AMP, ampicillin; HLG, high-level gentamicin; CIP, ciprofloxacin; NOR, norfloxacin; STR, streptomycin; PEN, penicillin G; TET, tetracycline; KF, cephalothin; NA, nalidixic acid; SXT, cotrimoxazole; CPD, cefpodoxime; CHL, chloramphenicol; VAN, vancomycin; ERY, erythromycin. Antibiotic susceptibility was determined using the disc diffusion method and the interpretation of antibiotic susceptibility was based on CLSI guidelines: Results are expressed as S = susceptible, I = intermediate, and R = resistant. Nevertheless, the observed profiles provide a useful comparative assessment of biosafety among the selected isolates.

Table S5 Carbohydrate utilization profile of LAB isolates determined by API 50 CHL system (bioMérieux SA, France)

No.	Code	Carbohydrate source	BP-2	BP-3	BPW-B1
0	CTRL	Control	–	–	–
1	GLY	Glycerol	–	–	–
2	ERY	Erythritol	–	–	–
3	DARA	D-Arabinose	–	–	–
4	LARA	L-Arabinose	–	–	–
5	RIB	Ribose	–	–	–
6	DXYL	D-Xylose	–	–	–
7	LXYL	L-Xylose	–	–	–
8	ADO	D-Adonitol	–	–	–
9	MDX	Methyl-β-D-xylopyranoside	–	–	–
10	GAL	Galactose	–	–	–
11	GLU	Glucose	+	+	+
12	FRU	Fructose	+	+	+
13	MNE	Mannose	–	–	–
14	SBE	Sorbose	–	–	–
15	RHA	Rhamnose	–	–	–
16	DUL	Dulcitol	–	–	–
17	INO	Inositol	–	–	–
18	MAN	Mannitol	+	+	+
19	SOR	Sorbitol	–	–	–
20	MDM	Methyl-α-D-mannopyranoside	–	–	–
21	MDG	Methyl-α-D-glucopyranoside	–	–	–
22	NAG	N-acetyl-glucosamine	–	–	–
23	AMY	Amygdalin	–	–	–
24	ARB	Arbutin	–	–	–
25	ESC	Esculin	+	+	+
26	SAL	Salicin	–	–	–
27	CEL	Cellobiose	–	–	–
28	MAL	Maltose	–	–	–
29	LAC	Lactose	–	–	–
30	MEL	Melibiose	–	–	–
31	SAC	Sucrose	+	+	+
32	TRE	Trehalose	+	+	+
33	INU	Inulin	–	–	–
34	MLZ	Melezitose	–	–	–
35	RAF	Raffinose	–	–	+
36	AMD	Starch	–	–	–
37	GLYG	Glycogen	–	–	–
38	XLT	Xylitol	–	–	–
39	GEN	Gentiobiose	–	–	–
40	TUR	Turanose	–	–	–

No.	Code	Carbohydrate source (cont.)	BP-2	BP-3	BPW-B1
41	LYX	Lyxose	–	–	–
42	TAG	Tagatose	–	–	–
43	DFUC	D-Fucose	–	–	–
44	LFUC	L-Fucose	–	–	–
45	DARL	D-Arabitol	–	–	–
46	LARL	L-Arabitol	–	–	–
47	GNT	Potassium gluconate	+	+	+
48	2KG	2-ketogluconate	–	–	–
49	5KG	5-ketogluconate	–	–	–

Note: (+) positive reaction; (–) negative reaction

Table S6 Enzymatic profiles of LAB isolates determined using API ZYM system (bioMérieux SA, France)

No.	Enzyme	BP-2	BP-3	BPW-B1
1	Control	–	–	–
2	Alkaline phosphatase	–	–	–
3	Esterase (C4)	–	–	–
4	Esterase lipase (C8)	–	–	–
5	Lipase (C14)	–	–	–
6	Leucine arylamidase	+	+	+
7	Valine arylamidase	–	–	–
8	Cystine arylamidase	–	–	–
9	Trypsin	–	–	–
10	α-chymotrypsin	–	–	–
11	Acid phosphatase	+	+	+
12	Naphthol-AS-BI-phosphohydrolase	+	+	+
13	α-galactosidase	–	–	–
14	β-galactosidase	–	–	–
15	β-glucuronidase	–	–	–
16	α-glucosidase	–	–	–
17	β-glucosidase	–	–	–
18	N-acetyl-β-glucosaminidase	–	–	–
19	α-mannosidase	–	–	–
20	α-fucosidase	–	–	–

Note: (+) positive enzymatic activity; (–) no detectable activity

Table S7 Amino acid profile of bee pollen fermented by osmophilic yeasts and probiotic LAB in single- and mixed-cultures

Amino acid	Amino acid content (μmol/mL)		
	[a]Yeast	[b]FP3	[c]FP123 LAB + yeast
Alanine	0.544 ± 0.028[b]	0.520 ± 0.026[b]	1.160 ± 0.058[a]
Cysteine	0.0106 ± 0.0007[b]	0.0094 ± 0.0006[b]	0.0161 ± 0.0010[a]
Aspartic acid	0.0361 ± 0.0021[ab]	0.0413 ± 0.0023[a]	0.0325 ± 0.0020[b]
Glutamic acid	0.3283 ± 0.016[b]	0.3079 ± 0.015[b]	0.4287 ± 0.020[a]
Phenylalanine	0.252 ± 0.013[b]	0.255 ± 0.014[b]	0.763 ± 0.038[a]
Glycine	0.185 ± 0.011[b]	0.175 ± 0.010[b]	0.527 ± 0.027[a]
Histidine	0.366 ± 0.018[b]	0.358 ± 0.019[b]	0.878 ± 0.044[a]
Isoleucine	0.0024 ± 0.0002[b]	0.0023 ± 0.0002[b]	0.0066 ± 0.0004[a]
Lysine	0.0684 ± 0.0041[b]	0.0165 ± 0.0012[c]	0.381 ± 0.019[a]
Leucine	0.0137 ± 0.0010[c]	0.152 ± 0.008[b]	0.791 ± 0.040[a]
Methionine	0.497 ± 0.025[b]	0.518 ± 0.026[b]	0.636 ± 0.031[a]
Proline	0.327 ± 0.017[b]	0.283 ± 0.016[b]	1.013 ± 0.051[a]
Arginine	1.022 ± 0.052[b]	0.929 ± 0.048[b]	2.633 ± 0.130[a]
Serine	0.228 ± 0.012[b]	0.215 ± 0.012[b]	0.480 ± 0.024[a]
Threonine	0.1582 ± 0.008[b]	0.1695 ± 0.009[a]	0.0461 ± 0.0030[c]
Tyrosine	0.0821 ± 0.004[b]	0.1141 ± 0.006[a]	0.0796 ± 0.0041[b]
Valine	0.249 ± 0.013[b]	0.221 ± 0.012[b]	0.470 ± 0.024[a]

[a]Yeast strains consisted of *Z. bailii* TSU_YK2 and *S. meliponinorum* TSU_YP10

[b] Single-strain LAB of *Apilactobacillus kunkeei* BPW-B1

[c] Multi-strain LAB of *A. kunkeei* BP-2, BP-3, and BPW-B1 and yeast *Z. bailii* TSU_YK2 and *S. meliponinorum* TSU_YP10

Differences among treatments were analyzed using one-way ANOVA followed by Tukey's HSD test ($p < 0.05$) ($n = 3$).

Supplementary Materials (Figure)

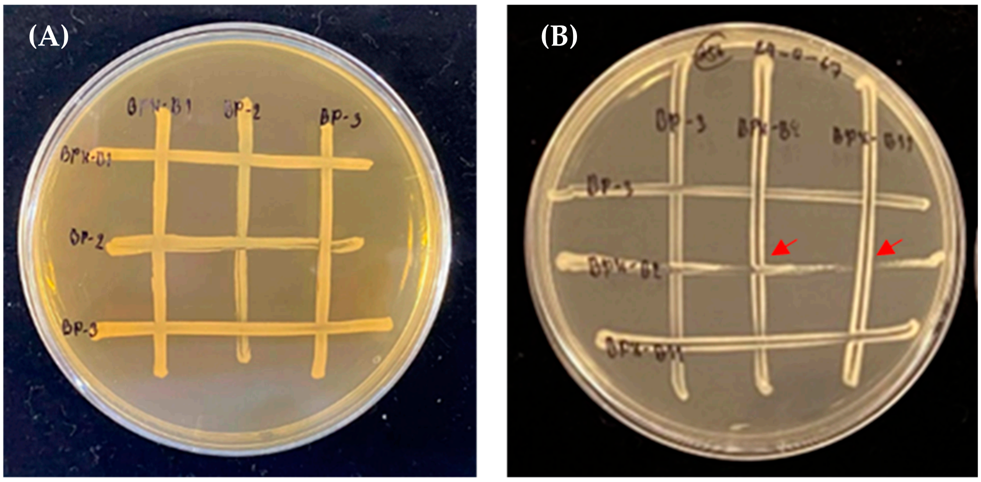

Indicator strain	BP-1	BP-2	BP-3	BPW-B1	BPW-B2
**BP-2**	+	–	+	+	±
**BP-3**	+	+	–	+	±
**BPW-B1**	+	+	+	–	±
**BPW-B2**	±	±	±	±	–
**BPW-B11**	±	±	±	±	±

(±) Partially compatible – slight reduction of growth density at intersection
(–) Self-control – same strain

Figure S1. Compatibility matrix of LAB isolates based on co-cultivation assay. Most LAB isolates demonstrated compatibility when co-cultivated, as indicated by normal growth at intersection zones. BP-2, BP-3, and BPW-B1 showed no antagonistic effects toward each other. In contrast, BPW-B2 and BPW-B11 exhibited partial growth inhibition (±), indicated by reduced growth density at intersection lines (red arrows), suggesting mild antagonistic interactions among certain strains.

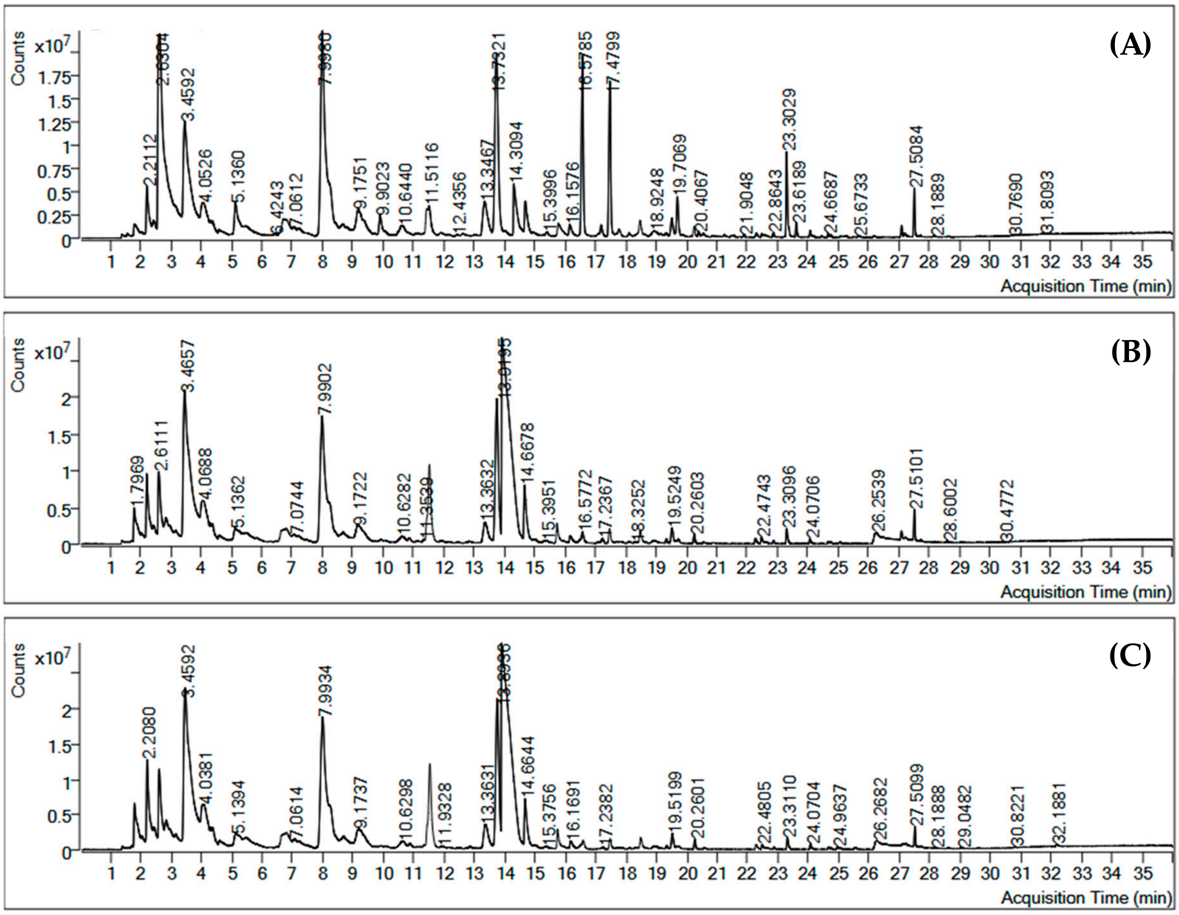

Figure S2. GC-MS chromatogram of distinct metabolite profiles in fermented bee pollen between yeast cultures: *Z. bailii* TSU_YK2 + *S. meliponinorum* TSU_YP10 (A) and *A. kunkeei* BPW-B1 and yeast (B), and mixed culture fermentation between FP123 and yeast (C). The mixed culture exhibited stronger peak intensity, particularly in the retention time range of 13–18 min, corresponding to organic acids and phenolic-related metabolites. The prominent peak at RT 13.89 min (acetic acid) was markedly higher in mixed culture, indicating enhanced carbohydrate utilization and increased metabolite synthesis during LAB–yeast co-fermentation.

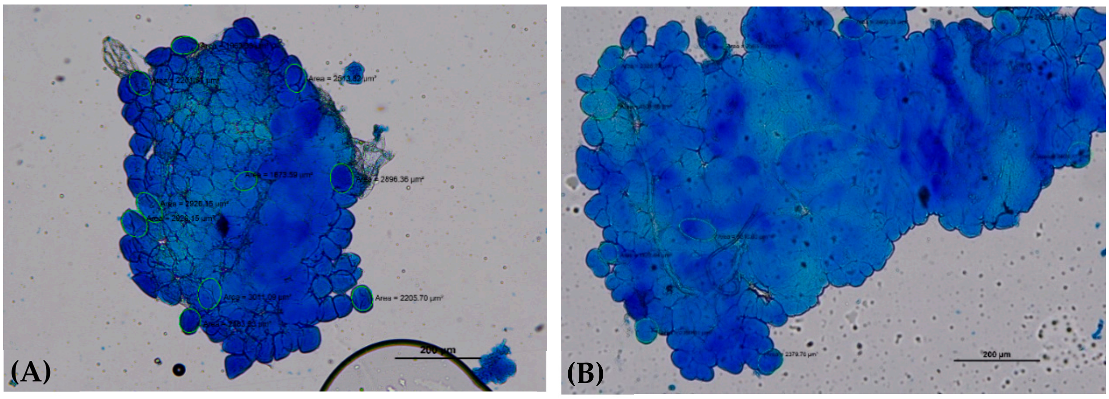

Figure S3. Hypopharyngeal glands (HPGs) of *Heterotrigona itama* workers after 7 days of feeding. Control (A; basal pollen) shows smaller acini compared to probiotic-fed bees (B), consistent with the larger mean acini size observed in probiotic-fed bees.

(A): without probiotic supplementation

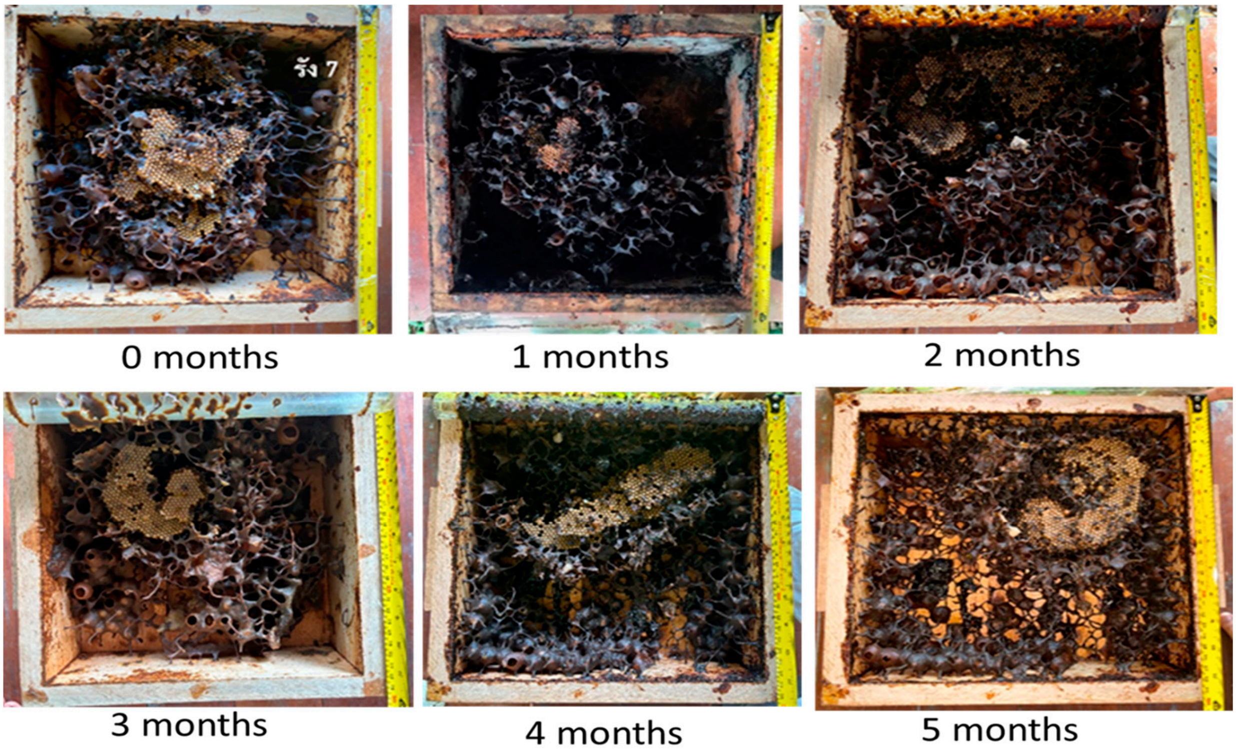

(B): probiotic-supplementation

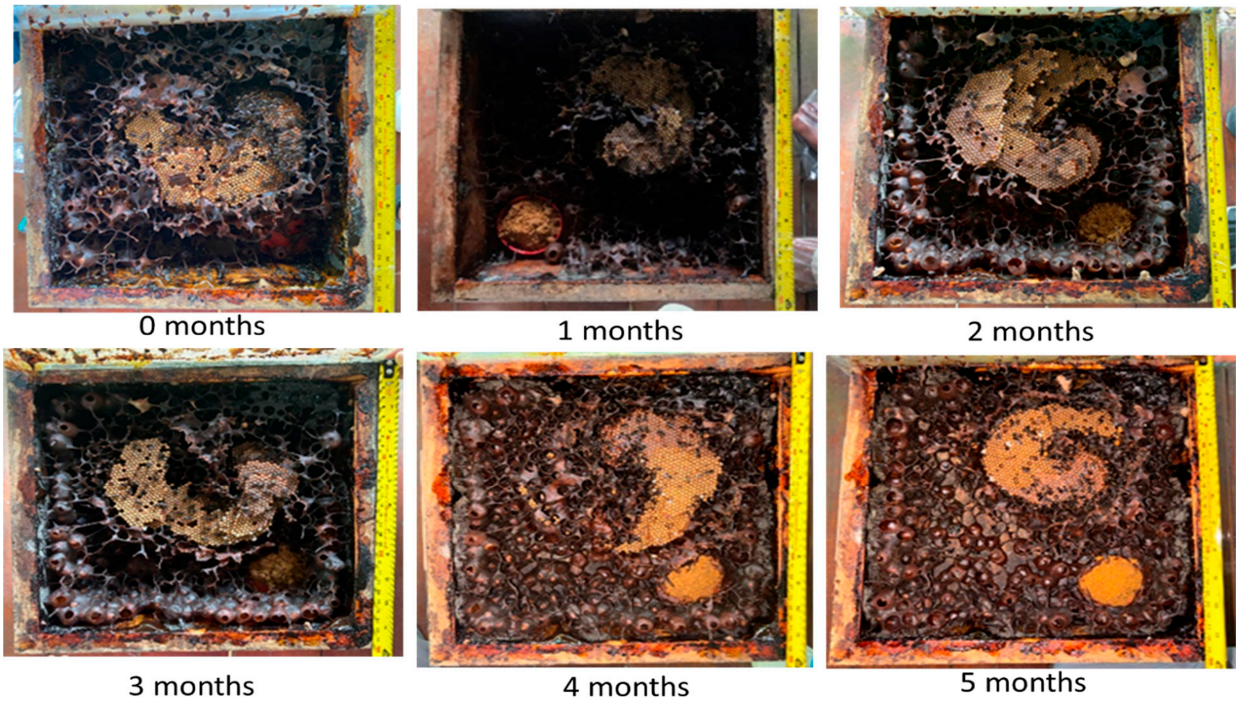

Figure S4. Comparison of the internal nest architecture of *Heterotrigona itama* colonies under different dietary treatments over a 6-month period. (A: control without probiotic supplementation; B: probiotic-supplemented).

During Months 0–1, both groups exhibited relatively small brood areas and limited honey storage pots. However, probiotic-supplemented colonies (B) showed more active brood development and a more organized nest structure than the control group (A). During Months 2–3, probiotic-fed colonies displayed greater brood expansion and denser nest organization, whereas control colonies developed more gradually. By Months 4–5, probiotic-supplemented colonies exhibited larger and more compact brood clusters together with more abundant honey storage pots than the control colonies. These observations suggest that probiotic supplementation was associated with enhanced colony development and resource storage in *H. itama* colonies.
